# Exploring Genetic Divergence in a Species-Rich Insect Genus Using 2790 DNA Barcodes

**DOI:** 10.1371/journal.pone.0138993

**Published:** 2015-09-25

**Authors:** Xiaolong Lin, Elisabeth Stur, Torbjørn Ekrem

**Affiliations:** Department of Natural History, NTNU University Museum, Norwegian University of Science and Technology, Trondheim, Norway; Consiglio Nazionale delle Ricerche (CNR), ITALY

## Abstract

DNA barcoding using a fragment of the mitochondrial cytochrome *c* oxidase subunit 1 gene (COI) has proven to be successful for species-level identification in many animal groups. However, most studies have been focused on relatively small datasets or on large datasets of taxonomically high-ranked groups. We explore the quality of DNA barcodes to delimit species in the diverse chironomid genus *Tanytarsus* (Diptera: Chironomidae) by using different analytical tools. The genus *Tanytarsus* is the most species-rich taxon of tribe Tanytarsini (Diptera: Chironomidae) with more than 400 species worldwide, some of which can be notoriously difficult to identify to species-level using morphology. Our dataset, based on sequences generated from own material and publicly available data in BOLD, consist of 2790 DNA barcodes with a fragment length of at least 500 base pairs. A neighbor joining tree of this dataset comprises 131 well separated clusters representing 121 morphological species of *Tanytarsus*: 77 named, 16 unnamed and 28 unidentified theoretical species. For our geographically widespread dataset, DNA barcodes unambiguously discriminate 94.6% of the *Tanytarsus* species recognized through prior morphological study. Deep intraspecific divergences exist in some species complexes, and need further taxonomic studies using appropriate nuclear markers as well as morphological and ecological data to be resolved. The DNA barcodes cluster into 120–242 molecular operational taxonomic units (OTUs) depending on whether Objective Clustering, Automatic Barcode Gap Discovery (ABGD), Generalized Mixed Yule Coalescent model (GMYC), Poisson Tree Process (PTP), subjective evaluation of the neighbor joining tree or Barcode Index Numbers (BINs) are used. We suggest that a 4–5% threshold is appropriate to delineate species of *Tanytarsus* non-biting midges.

## Introduction

**Fig 1:**
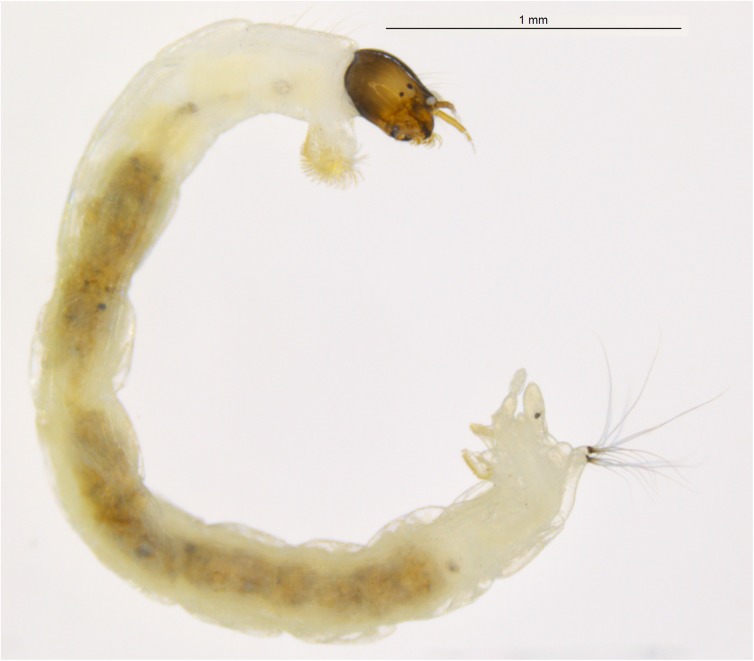
*Tanytarsus* sp.10XL, larva.

Genetic variation between species in cytochrome c oxidase subunit 1 (COI) gene sequences has been proven informative for species identification in many animal taxa, including non-biting midges, the Chironomidae (Insecta, Diptera) [1–5]. The mutation rate in COI can be fast enough to provide informative characters for delineation of closely related and sibling species and even to analyze phylogeographic patterns within a single species [6–9]. Many chironomid species, especially in the larval life stage (Fig 1), are difficult to identify and partial COI gene sequences as DNA barcodes have been shown appropriate to delimit and identify species as well as associate life stages in this family [3, 10–14]. In general, partial COI sequences show a high-level of divergence between species in Chironomidae, so high that the marker performs poorly in phylogenetic reconstructions [3, 15]. Nevertheless, COI has been used to infer the phylogenetic relationship within family Chironomidae [16, 17].

The dipteran family Chironomidae is the most ubiquitous and usually most abundant insect group in all types of freshwater and even saltwater [18]. At present, there are more than 6000 described species worldwide (P. Ashe pers comm.) and certain species can reach densities up to 15600 individuals per m^2^ at favorable conditions [19]. Due to their high abundance and diversity, chironomid larvae occupy a key position among benthic macroinvertebrates, they are important as freshwater indicator organisms [20] and as food items for fish. In addition, larval head capsules are preserved in lake sediments and have been shown to be useful in climate reconstructions since species composition varies with water temperature as well as other environmental factors. However, identification of chironomid larvae to species-level via morphology usually is arduous, time-consuming and expensive, and more effective identification techniques such as DNA barcoding can greatly improve the use of chironomids in biological-assessments of freshwater ecosystems [21–23]. Recently, this was exemplified with a next-generation sequencing protocol analyzing 1015 tropical chironomids at a cost of less than $1 per specimens [24]. At present, there are few publications that investigate the performance of DNA barcoding in species-rich genera. A couple of studies are known from plants [25–27], but there are currently no papers that explore how efficient DNA barcodes are in delineation of species in larger genera (>200 species) of insects.

**Fig 2:**
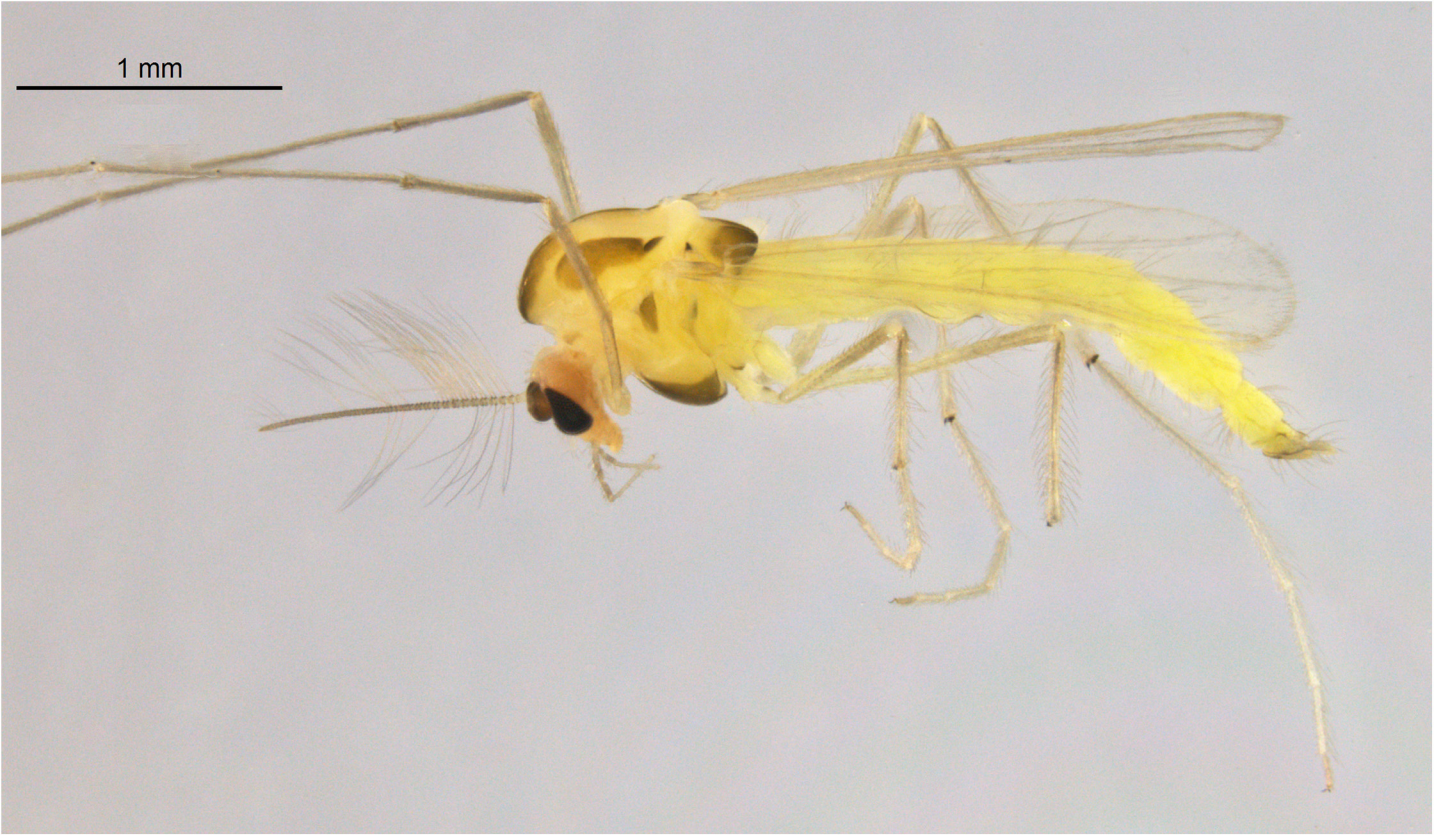
*Tanytarsus occultus* Brundin, 1949, adult male.

The genus *Tanytarsus* van der Wulp, 1874 is the most species-rich genus of the tribe Tanytarsini in subfamily Chironominae with more than 400 described species worldwide. Species of the genus *Tanytarsus* (Fig 2) are eurytopic, and immatures occur in all types of freshwater. There are even species with larvae and pupae in marine or terrestrial environments[28]. The genus was erected by van der Wulp [29] and various species groups and regionally distributed species have been revised over the last few decades [30–37]. A morphological determination of some *Tanytarsus* species group can be extremely challenging. Moreover, there are many unknown and cryptic species in *Tanytarsus* and it is difficult to associate the immature stages with adults through rearing since it is time-consuming and not always successful. In general, identification at the species-level strongly relies on the morphological characters of adult males. However, diagnostic characters might be unreliable due to intraspecific morphological variation even for this life stage and phenotypic plasticity [38] in morphometric ratios and hypopygial structures caused by different temperature regimes and food quality has been observed in several chironomid species [39]. Moreover, artifacts created in the slide-mounting process can also obscure species specific characteristics.

Currently, there are several approaches to analyze how DNA barcode data form separate genetic clusters potentially corresponding to biological species. In this context, evaluation of neighbor joining [40] ID-trees perhaps represents the most widely used method for the direct comparison of DNA barcodes. In general, neighbor joining trees are easy and fast to compute with appropriate bootstraps replicates even for big datasets. Furthermore, methods such as Automatic Barcode Gap Discovery (ABGD) [41], the Generalized Mixed Yule Coalescent model (GMYC) [42–44], the Poisson Tree Process (PTP) [45], Objective Clustering [46], and the Barcode Index Numbers Algorithm (BINs) [47] also have been proven to represent effective approaches to group hypothetical species in a sequence alignment. ABGD aims to assign sequences into Operational Taxonomic Units (OTUs) based on a statistically inferred barcode gap in an initial partitioning, conducting a second round of splitting through recursive partitioning [41, 48]. ABGD performs well for standard prior maximum intraspecific divergences except for datasets including less than three sequences per species [41]. The GMYC model is a likelihood method for delimiting species by fitting within and between species branching models to gene trees [43] while the Poisson Tree Process (PTP) is a method for species delimitation based on rooted phylogenetic trees [45]. The PTP-model assumes that intra- and interspecific substitutions follow two distinct Poisson processes, and that intraspecific substitutions are discernibly fewer than interspecific substitutions [44]. The Objective Clustering method of the software Species Identifier aims to explore intra- and interspecific genetic distances of cluster sequences based on pairwise distances [46]. Species Identifier allows the comparison of clusters generated using preset thresholds by users with existing taxonomy [49–51]. The Barcode Index Numbers Algorithm (BIN) is incorporated in the Barcode of Life Data Systems (BOLD, www.boldsystems.org) [47, 52]. BIN analysis generates one number of OTUs for each set of DNA barcode sequences using the Refined Single Linkage algorithm [48]. The BIN algorithm has been effectively tested on numerous taxonomic groups and shows potential for applications in species abundance studies and environmental barcoding [47]. Nevertheless, few studies have compared the performance of the novel analytical methods for the DNA barcode-based delineation of OTUs [48].

In this study, we have used 2790 *Tanytarsus* DNA barcodes to test the utility of COI barcodes for species identification in *Tanytarsus*. Furthermore, we used DNA barcodes and morphology to evaluate potentially cryptic species within this genus. Finally, we compared the number of OTUs using ABGD, GMYC, PTP, Objective Clustering and the BIN algorithm to see which of these methods correspond best to morphological species concepts in *Tanytarsus* and which level of intraspecific divergence we should expect within this genus.

## Materials and Methods

### Taxon sampling and data collection

The *Tanytarsus* sequences used in this study originated from specimens that were collected in many different parts of the world. Own field work was conducted mainly in Northern Europe, China and Canada during recent years, but chironomids were also collected in Central Europe, North and Central America, Africa and Australia. Specimens were identified morphologically using taxonomic revisions and species description [31, 32, 34–36, 53–64].

In addition to own data, we searched for public COI barcodes in BOLD belonging to genus *Tanytarsus* that were longer than 500 base pairs and lacked stop codons, indicating absence of dysfunctional copies of mitochondrial genes (NUMTs). Searches were done January 17, 2015. Hits were combined with own data and are available through the dataset “*Tanytarsus* DNA barcoding 2015 (DS-TABAC)” on BOLD, DOI: dx.doi.org/10.5883/DS-TABAC.

The complete dataset includes 2790 COI sequences of which 164 originated from GenBank, 340 from our own lab at the Department of Natural History, NTNU University Museum, and the remaining 2286 sequences from various projects in BOLD. In our dataset, 1242 of 2790 specimens were not examined by us and only identified to genus-level. Since this would make analyses of intra- and interspecific distances difficult, we re-named the 1242 sequences by the clusters they belonged to in a standard neighbor joining tree based on Kimura 2-Parameter (K2P) [65] distances (S1 File). In cases where unidentified sequences matched named morphospecies, we gave them this name; in cases where there was no matching morphospecies name, we gave all sequences in that cluster the same distinguishable group name.

### DNA extraction, PCR amplification, sequencing and alignment

Sampled specimens were preserved in 75–96% ethanol and stored dark at 4°C before molecular analyses. Depending on size, a single or three legs were removed from the majority of specimens and sent to Canadian Centre for DNA Barcoding, the Biodiversity Institute of Ontario (Guelph, Ontario, Canada) for DNA extraction, PCR and bi-directional Sanger sequencing as part of the International Barcode of Life project. In addition, DNA of 102 specimens was extracted from the thorax and head using GeneMole DNA Tissue Kit on a GeneMole^®^ instrument (Mole Genetics, Lysaker, Norway) at the Department of Natural History, NTNU University Museum. The standard protocol was followed with exception that 4 μl Proteinase K was mixed with 100 μl buffer for overnight lysis at 56°C. The final elution volume was 100 μl. After DNA extraction, the exoskeleton was washed with 96% ethanol and mounted in Euparal on the same microscope slide as its corresponding antennae, wings, legs and abdomen following the procedure outlined by Sæther [66]. Vouchers are deposited at the Department of Natural History, NTNU University Museum, Trondheim, Norway and College of Life Sciences, Nankai University, Tianjin, China (Chinese specimens).

A 658 bp fragment of the COI region was PCR-amplified using the universal primers LCO1490 and HCO2198 [67]. DNA amplification was carried out in 25 μl reactions using 2.5 μl 10x Takara ExTaq pcr buffer (CL), 2 μl 2.5 mM dNTP mix, 2 μl 25 mM MgCl_2_, 0.2 μl Takara Ex Taq HS, 1 μl 10 μM of each primer, 2 μl template DNA and 14.3 μl ddH_2_O. Amplification cycles were performed on a Biorad C1000 Thermal Cycler (Bio-Rad, California, USA) and followed a program with an initial denaturation step of 95°C for 5 min, then followed by 34 cycles of 94°C for 30 s, 51°C for 30 s, 72°C for 1 min and 1 final extension at 72°C for 3 min. PCR products were purified using illustra ExoProStar 1-Step (GE Healthcare Life Sciences, Buckinghamshire, UK) and shipped to MWG Eurofins (Ebersberg, Germany) for bidirectional sequencing using BigDye 3.1 (Applied Biosystems, Foster City, CA, USA) termination.

Sequences were assembled and edited using Sequencher 4.8 (Gene Codes Corp., Ann Arbor, Michigan, USA). Sequence information was uploaded on BOLD (www.boldsystems.org) along with an image and collateral information for each voucher specimen.

The sequences names were edited using MESQUITE 2.50 [68]. Alignment of the sequences was carried out using the Muscle algorithm [69] on amino acids in MEGA 6 [70] (S2 File).

The nucleotide statistics and pairwise distances using the K2P model were calculated in MEGA 6 (S1 Table). The neighbor joining tree was conducted using K2P substitution model with 500 bootstrap replications and the “pairwise deletion” option of missing data in MEGA 6. The K2P model was used to make our results comparable with most other DNA barcode studies on insects.

To estimate the number of OTUs, the aligned sequences were subjected to Objective Clustering at 2–7% threshold in Species Identifier (TaxonDNA 1.6.2) [46]. In addition, the aligned sequences were sorted into hypothetical species using ABGD method with a prior *P* that ranges from 0.005 to 0.1, and the K2P model, following the default settings. The number of BINs in the dataset was counted as they appeared in BOLD on March 28^th^, 2015. Furthermore, a reduced dataset with 1250 unique sequences (haplotypes) was generated using ElimDupes (https://hcv.lanl.gov/content/sequence/ELIMDUPES/elimdupes.html) and manual inspection for use in both GMYC and PTP. The ultrametric tree required for the GMYC method was obtained using BEAST 1.8 [71] on the reduced dataset. The MCMC chain was run for 50 million generations under the HKY substitution model with two partitions (positions 1+2; position 3) and the Yule speciation model. Runs using more complex and fit models of substitution (e.g. GTR+I+G) was also attempted, but MCMC failed to start in BEAST due to low initial likelihoods even with UPGMA and ML starting trees. Prior settings are available from the authors. The MCMC log on prior and posterior values was examined in Tracer 1.6 [72] and a burn-in of 10 million generations was used to avoid suboptimal trees in the final consensus tree. The single-threshold GMYC method was applied using the *splits* package [73] in R [74] with step-by-step guides available on Tomochika Fujisawa’s blog (https://tmfujis.wordpress.com/2013/04/23/how-to-run-gmyc/). For PTP-based OTU estimation, the needed rooted phylogenetic input-tree was constructed with RAxML [75] using raxmlGUI v1.3 [76] with the GTR+G+I substitution model. The PTP model was implemented following the default parameters and 500 000 generations on the bPTP web server (http://species.h-its.org/ptp/) [45] as well as 1 000 000 generations on the stand-alone version in a Linux environment.

## Results and Discussion

### Barcode analysis

The aligned 2790 sequences ranged from 507 to 658 base pairs, including 798 sequences with full barcode length. In total, there were 338 variable sites (51.4%), of which 301 (89.1%) were parsimony informative. Most variable sites occurred in the third codon-position. The sequences were heavily AT-biased specifically in the third position with an average AT-composition of 87.6% (Table 1).

**Table 1:** Variable and informative sites, and average nucleotide composition in the aligned COI gene sequences.

Nucleotide Position	Variable Site (%)	Informative Site (%)	T (%)	C (%)	A (%)	G (%)	AT (%)	GC (%)
1st	25.7	22.6	26	17.3	29.2	27.4	55.2	44.7
2nd	9.2	4.9	43	27.2	13.3	16.5	56.3	43.7
3rd	65.1	72.4	45	8.9	42.6	3.1	87.6	12.4
All	51.4	89.1	38.1	17.8	28.4	15.7	66.5	33.5

**Fig 3:**
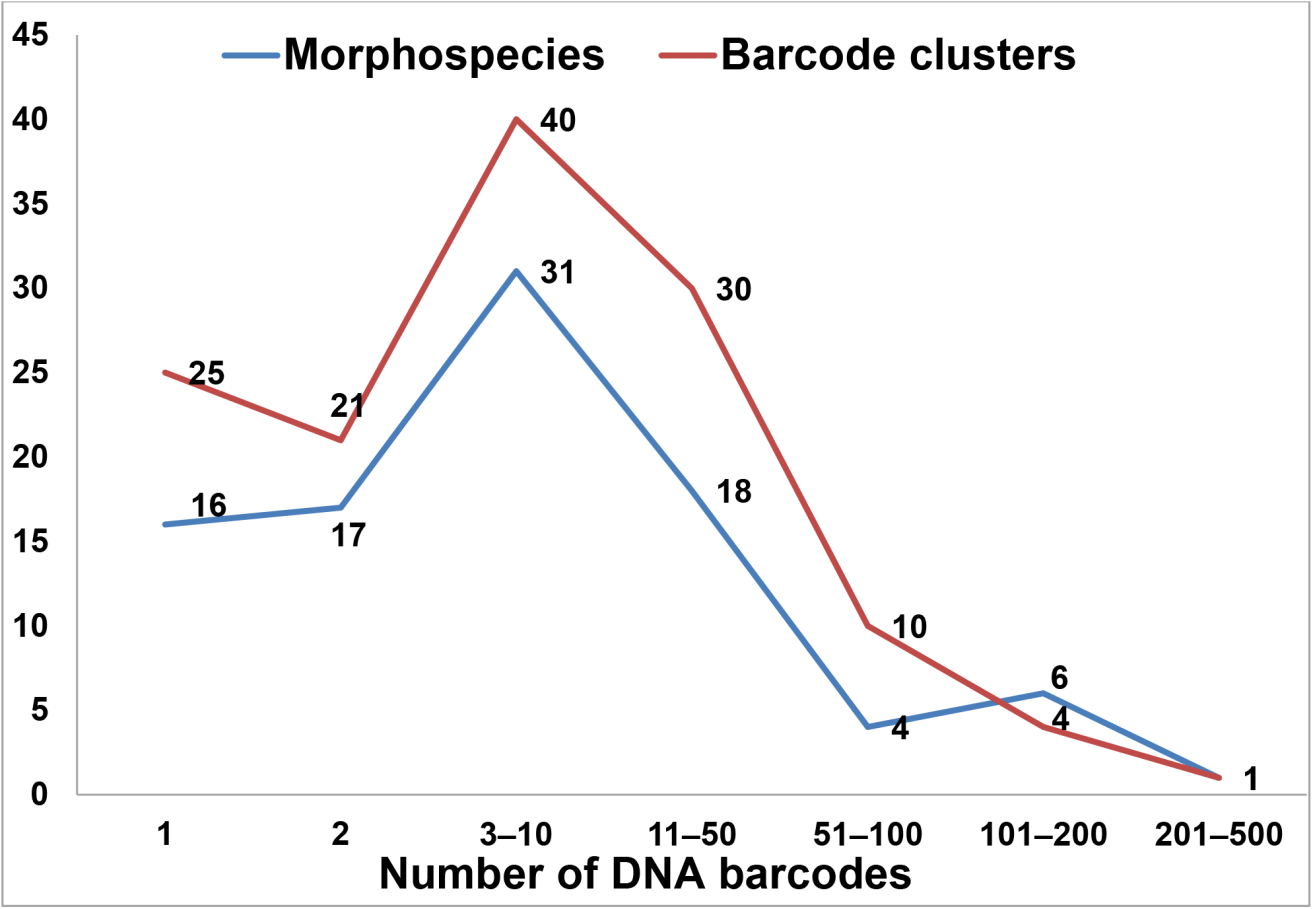
Number of DNA barcodes per morphospecies and barcode clusters based on the neighbor joining tree.

Our dataset included 1548 barcode sequences which before analysis were identified to species-level and 1242 barcodes which were identified to the genus-level. The number of DNA barcodes per morphospecies (n = 93) and DNA barcode cluster (n = 131) ranged from 1 to 430 (Fig 3).

Average intraspecific divergence was 2.14% (S2 Table) with maximum intraspecific divergence observed in *Tanytarsus brundini* Lindeberg (21.1%). This was even beyond the average interspecific divergence (15.9%) and sequences belonging to this morphospecies clustered clearly in four genetically divergent groups, indicating cryptic species. A similar situation was also observed for other morphospecies (see below). When disregarding obvious cryptic species complexes, the maximum intraspecific divergence was 8.5% (for *Tanytarsus occultus* Brundin). The maximum interspecific divergence was 26.7% between *Tanytarsus mendax* Kieffer and *Tanytarsus nigricollis* Goetghebuer. The minimum interspecific divergence was 0.9% between *Tanytarsus unagiseptimus* Sasa and *Tanytarsus kiseogi* Ree & Jeong, but this case probably was due to a misidentification of specimens not available to us for morphological examination and might also indicate a taxonomic synonym (see below).

**Fig 4:**
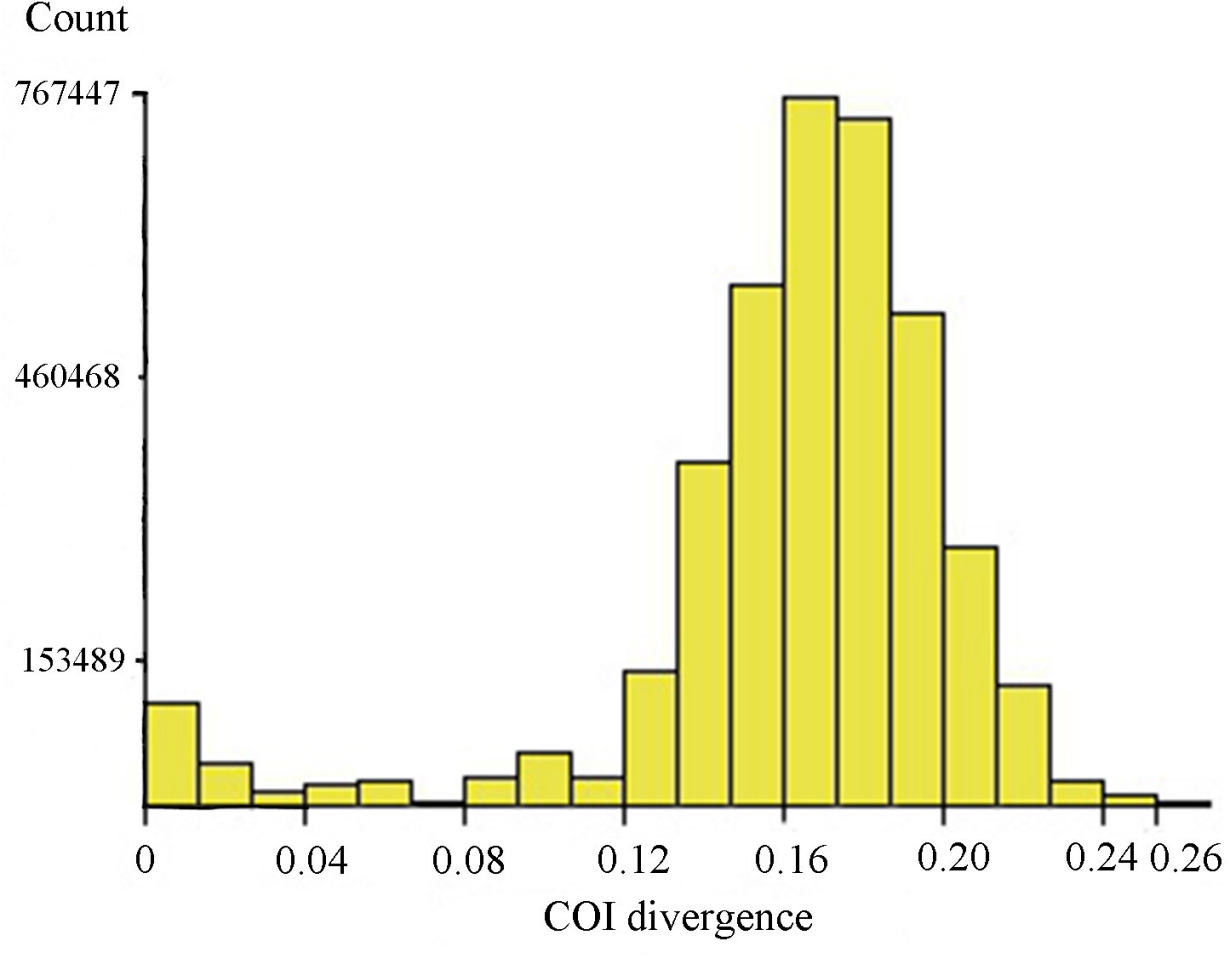
Histogram of pairwise K2P distances of 2790 aligned sequences. The figure was a result of analysis with ABGD using the K2P model. The horizontal axis shows the pairwise K2P-distance, and the vertical axis shows the number of pairwise sequence comparisons.

**Fig 5:**
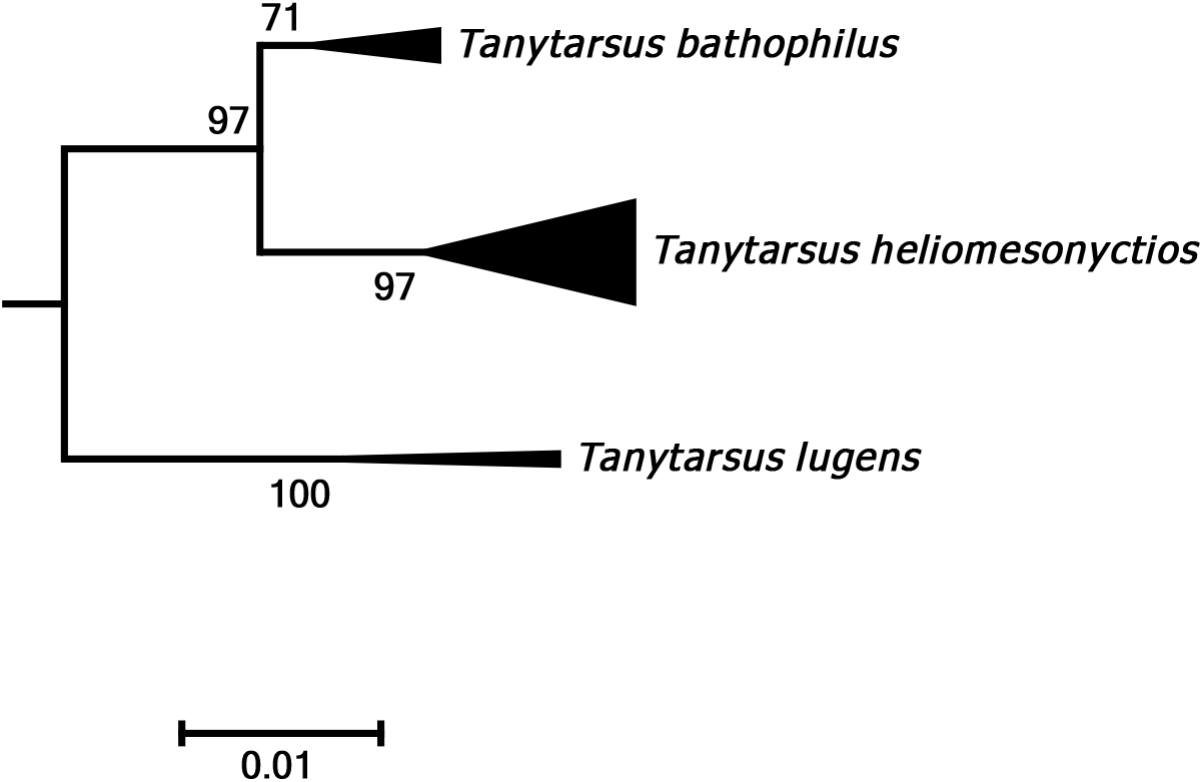
Neighbor joining subtree of the *Tanytarsus lugens* species group. Numbers on branches represent bootstrap support (>70%) based on 500 replicates; scale represents K2P genetic distance.

In general, our data showed distinctly larger interspecific (S3 Table) than intraspecific divergences, but due to the presence of cryptic species diversity and a few misidentifications, there was no clear “barcode gap” in the pairwise K2P distances (Fig 4). In addition, there are some cases of low genetic divergence between morphologically distinguishable species, likely due to recent speciation. For instance, three related morphospecies in the *lugens* species group, *Tanytarsus lugens* Kieffer, *Tanytarsus bathophilus* Kieffer and *Tanytarsus heliomesonyctios* Langton cannot be well-differentiated by DNA barcodes having interspecific pairwise distance up to about 3.2% (Fig 5).

### Species discrimination

The neighbor joining tree based on 2790 DNA barcodes comprises 131 well separated clusters, representing 77 named and 44 unnamed morphological species of *Tanytarsus* (S1 File). Among these unnamed species, 16 identified morphological species might be new to science, while 28 barcode clusters were not otherwise assignable to valid morphospecies. The results showed that DNA barcode clusters in general corresponded well with morphological species concepts in *Tanytarsus*; 94.6% (88/93) of the species identified based on morphology matched divergent barcode clusters.

However, DNA barcodes were not sufficient for identification in all cases. Previous studies have shown that the presences of NUMTs [77–79], symbiotic bacteria [80], incomplete lineage sorting [81–83], introgression [84, 85] and distant geographic areas can present obstacles in species delimitation [86, 87] using DNA barcoding. NUMTs and symbiotic bacteria, like *Wolbachia* have to our knowledge not yet been recorded in Chironomidae, but the three other causes are possible explanations for the observed inconsistencies between morphological and molecular species clusters.

A few examples of deep COI sequence divergence among specimens assigned to a single morphospecies were detected. There were at least two divergent barcode clusters in *Tanytarsus aterrimus* Freeman, *Tanytarsus bathophilus* Kieffer, *Tanytarsus brundini*, *Tanytarsus glabrescens* Edwards, *Tanytarsus guerlus* (Roback), *Tanytarsus heusdensis* Goetghebuer, *T*. *lestagei* Lindeberg, *Tanytarsus occultus*, *Tanytarsus takahashii* Kawai & Sasa and *Tanytarsus telmaticus* Lindeberg (S1 File).

**Fig 6:**
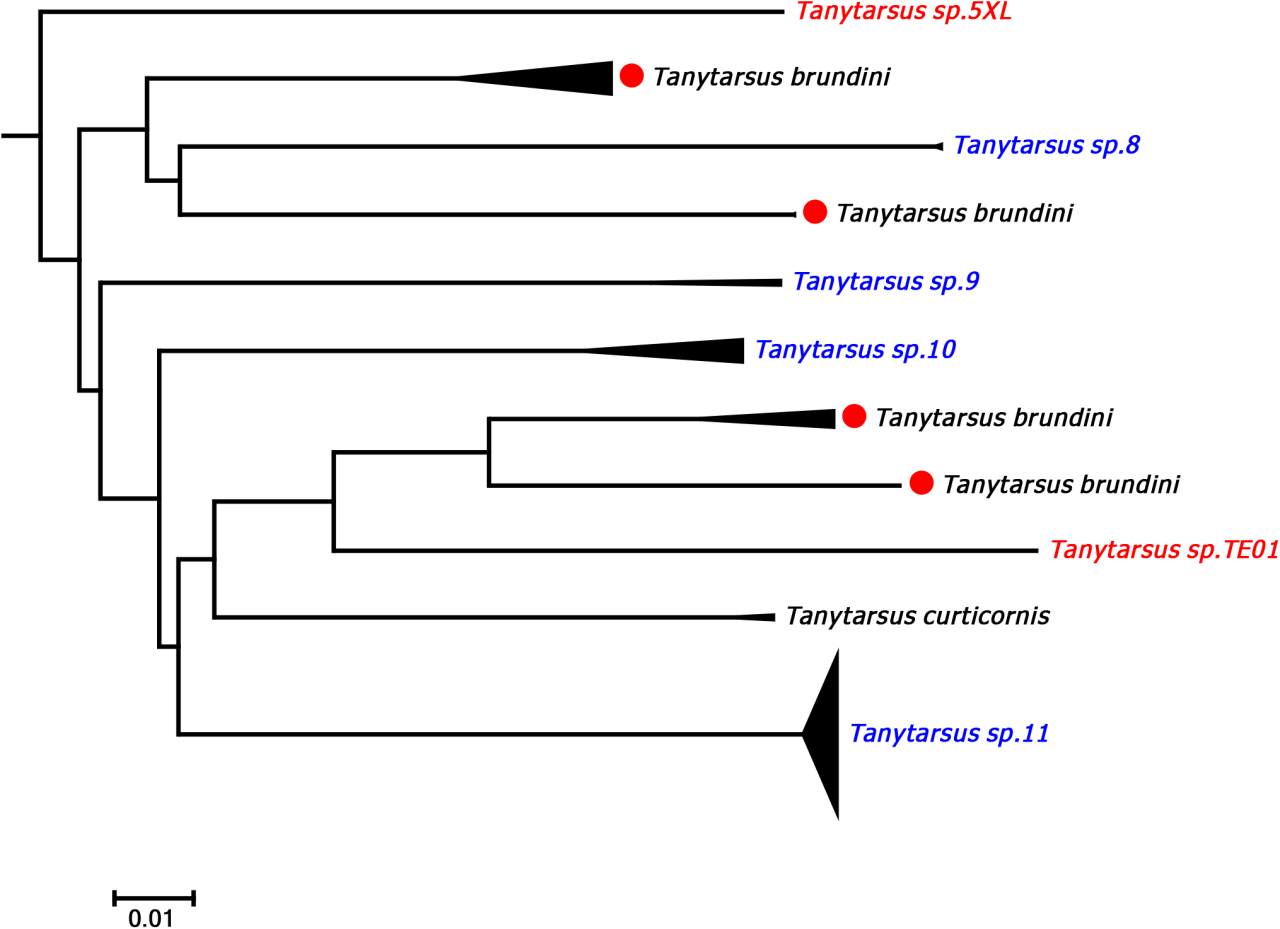
Neighbor joining subtree of the *Tanytarsus brundini* species complex. Numbers on branches represent bootstrap support (>70%) based on 500 replicates; scale represents K2P genetic distance.

For *T*. *brundini*, intraspecific pairwise K2P distances ranged from 0 to 21.1% and a total of four well separated barcode clusters Western Europe and Canada were observed (Fig 6). Examination of the voucher specimens did not reveal any distinct morphological characters corresponding with the clustering in COI sequences although some zoogeographical structure is present. We therefore suspect that this morphological species contains several cryptic species. A similar situation is present in *T*. *heusdensis*, another member of the *Tanytarsus chinyensis* Goetghebuer species group. There were three distinct barcode clusters of *T*. *heusdensis* in the result from our analyses, but no obvious morphological characters that will separate adult males from Germany and Norway (S1 File). Thus, it appears that geographically separated populations of some species in the *T*. *chinyensis* group are genetically divergent, but difficult to separate based on morphology. Identification of species in this group has also previously been acknowledged as challenging [36, 55, 57], thus it is perhaps not surprising that hidden genetic diversity is detected among members in the *T*. *chinyensis* group.

Members of the South African *T*. *aterrimus* also showed high intraspecific divergences with pairwise K2P-distances of up to 13.9%. The DNA barcodes clustered into 3 groups (S1 File) that so far could not be differentiated via morphology.

**Fig 7:**
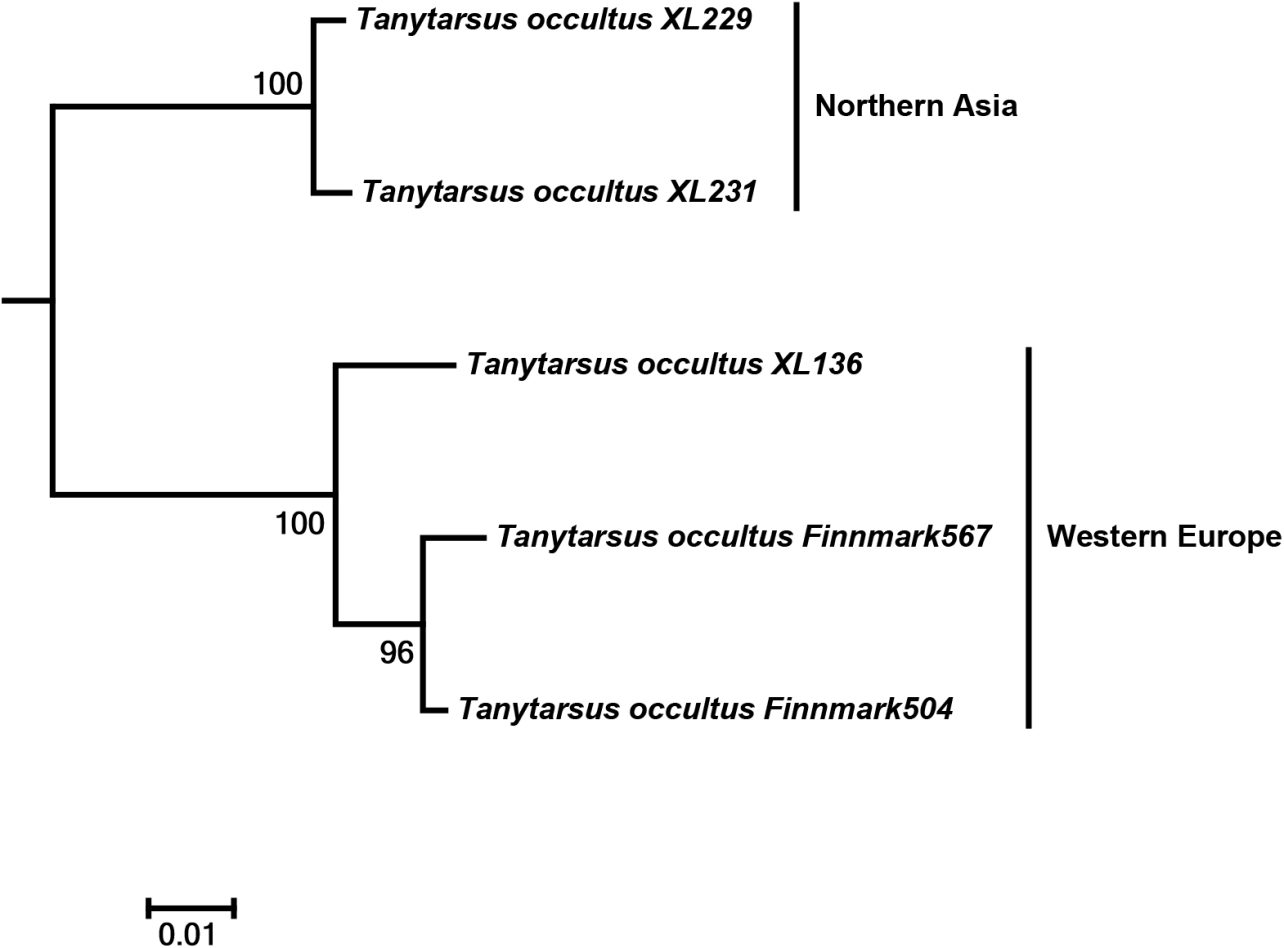
Neighbor joining subtree of *Tanytarsus occultus*. Numbers on branches represent bootstrap support (>70%) based on 500 replicates; scale represents K2P genetic distance.

**Fig 8:**
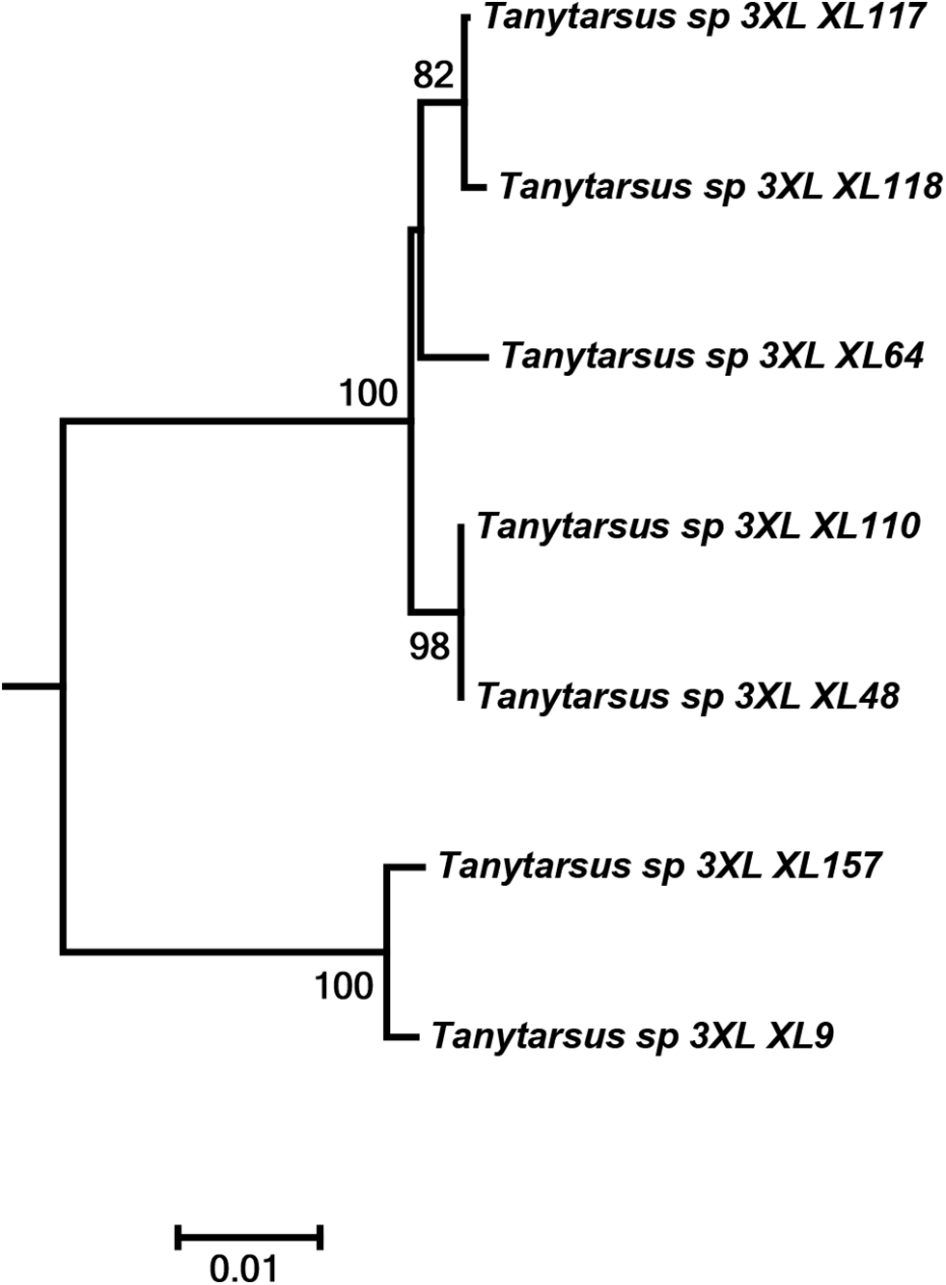
Neighbor joining subtree of *Tanytarsu*s sp.3XL from Tibet, China. Numbers on branches represent bootstrap support (>70%) based on 500 replicates; scale represents K2P genetic distance.

Another interesting case was observed in *T*. *occultus* where specimens from Northeastern Asia and Western Europe separated into two distinct clusters with sequence divergences from 7% to 8.5% (Fig 7). The adult male vouchers examined are as far as we can observe at present morphologically indistinguishable. Also, DNA barcodes of a hitherto undescribed morphospecies from Tibet, *Tanytarsus* sp.3XL, showed high intraspecific divergences and might be more than one species (Fig 8).

**Fig 9:**
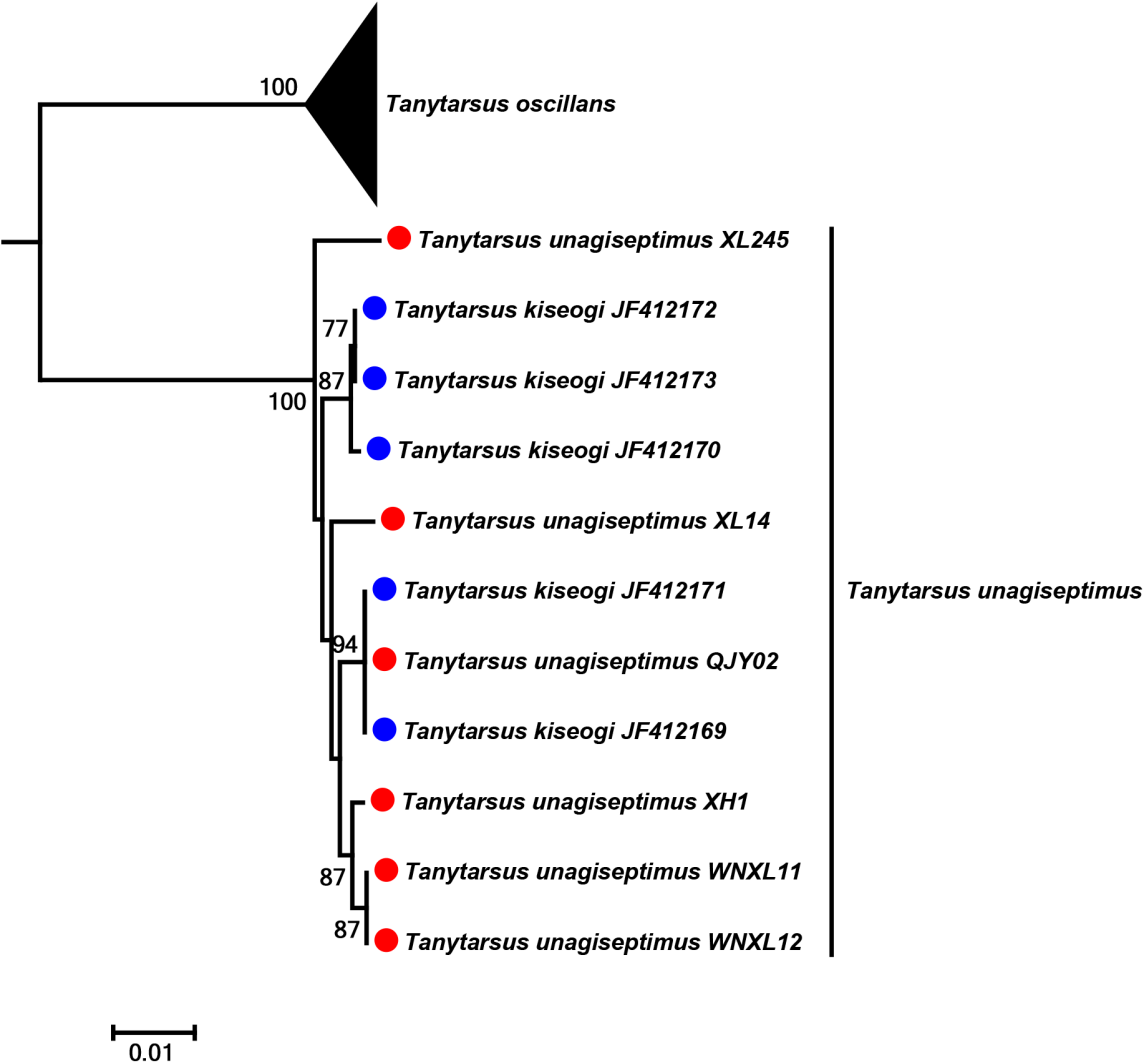
Neighbor joining subtree of *Tanytarsus kiseogi*, *Tanytarsus oscillans* and *Tanytarsus unagiseptimus*. Numbers on branches represent bootstrap support (>70%) based on 500 replicates; scale represents K2P genetic distance.

In addition to this previously undetected diversity, our results also suggest some new taxonomic synonyms on the species-level. For example, three closely related species in *Tanytarsus eminulus* species group, *Tanytarsus oscillans* Johannsen, *T*. *unagiseptimus* and *T*. *kiseogi*, distributed in China, Japan, South Korea, the Russian Fast East and Singapore, are differentiated by subtle morphological differences in the adult male genitalia [32, 60]. DNA barcode data indicate that *T*. *kiseogi* should be regarded as a junior synonym of *T*. *unagiseptimus* as the maximum interspecific divergence between specimens of these species was 1.5%. It is not clear if the *T*. *kiseogi* specimens from which the COI-sequences in GenBank originates are part of the type material, but they were possibly identified by one of the authors from the original species description since he is co-authoring the DNA barcode paper that published these sequences. However, a formal synonymy should await comparison of type material. *Tanytarsus oscillans* and *Tanytarsus unagiseptimus*, on the other hand, probably are two valid species with a minimum interspecific divergence at 7% (Fig 9). In this case, the subtle morphological difference (i.e. extensively distributed microtrichia between the crests of the anal point in *T*. *oscillans* compared to a smooth surface in *T*. *unagiseptimus*) was perfectly mirrored by COI divergence.

**Fig 10:**
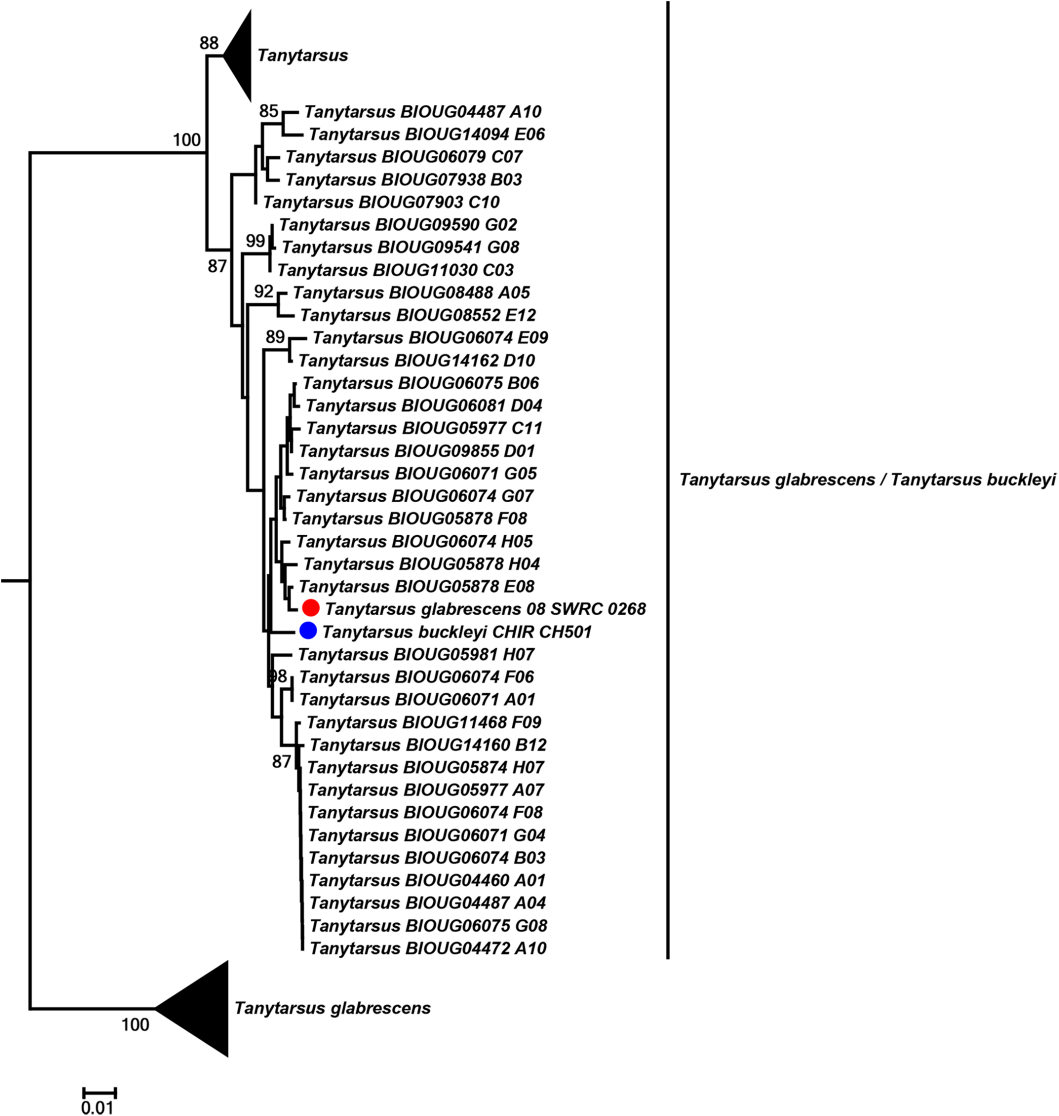
Neighbor joining subtree of *Tanytarsus buckleyi* and *Tanytarsus glabrescens*. Numbers on branches represent bootstrap support (>70%) based on 500 replicates; the dots indicate the specimens identified morphologically; scale represents K2P genetic distance.

Some mismatches between barcode clusters and identifications might be a result of misidentifications or differences in opinion between identifiers. This is to be expected because there are several groups in *Tanytarsus* with challenging taxonomy. Moreover, this type of mismatch can also occur if a reference database is used to identify unknown specimens but the identifications of these are not updated at the same time as the original reference sequences(s) if these change name. We found that *T*. *glabrescens* together with some unnamed *Tanytarsus* sequences grouped into three well-differentiated barcode clusters, which might demonstrate potential cryptic species within this species complex. One of the clusters was particularly interesting as single individuals of both *T*. *glabrescens* and *Tanytarsus buckleyi* Sublette were present together with many sequences from unidentified specimens (Fig 10). In case like this it is tempting to regard the one *T*. *glabrescens* as a misidentification since the species name already is present in two other clusters. However, it can only be clarified through examination of voucher specimens and collaboration between identifiers. It is therefore a great advantage for the taxonomy of challenging groups to deposit reference data in a database that facilitates communication between contributors and identifiers, such as BOLD, and voucher specimens of the sequences in an accessible collection [88].

**Fig 11:**
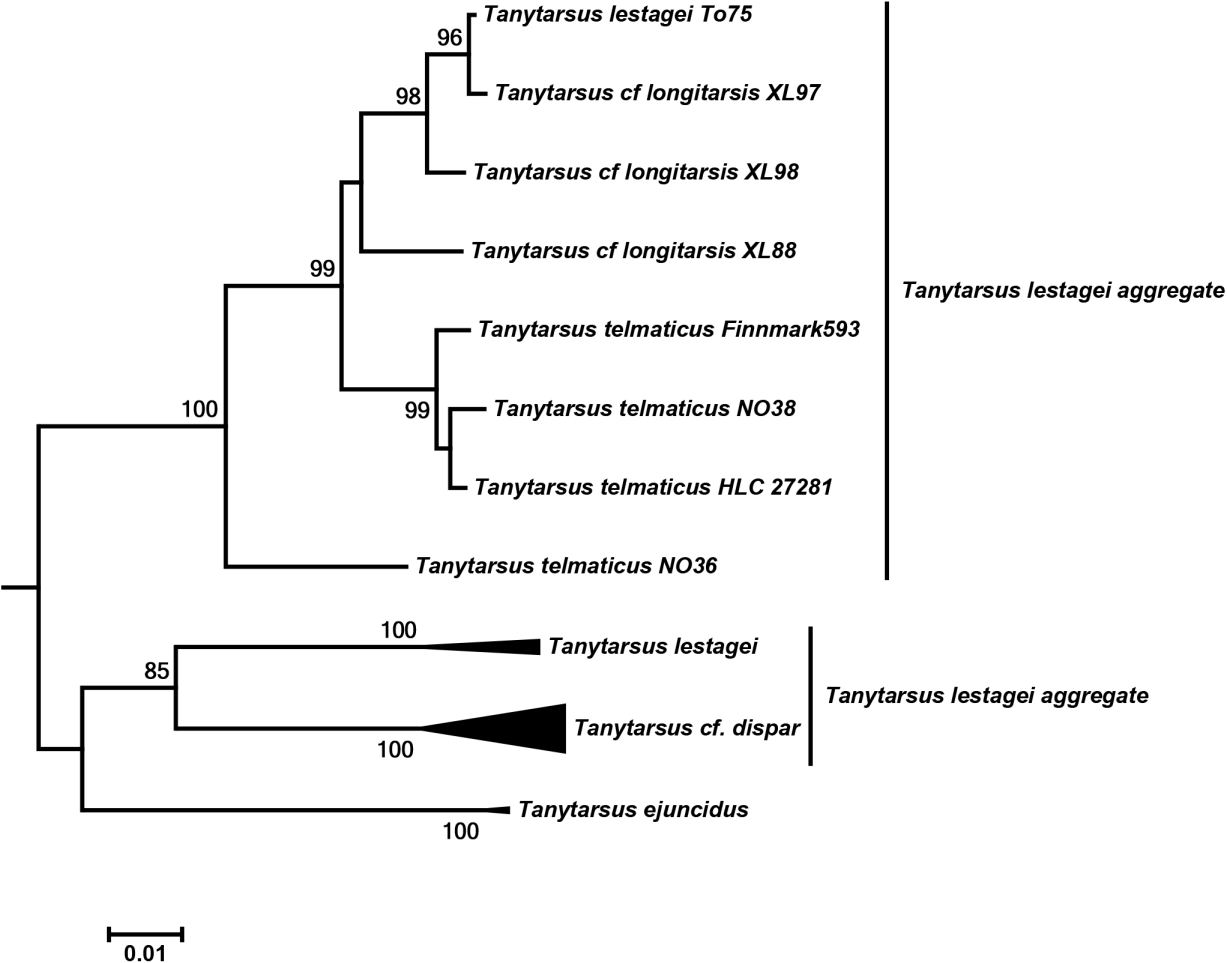
Neighbor joining subtree of European *Tanytarsus lestagei* aggregate. Numbers on branches represent bootstrap support (>70%) based on 500 replicates; scale represents K2P genetic distance.

A very similar situation was observed in the *Tanytarsus lestagei* aggregate which consists of several almost identical species [36, 58]. Within the European populations, some specimens of *T*. *lestagei*, *T*. *telmaticus and Tanytarsus* cf. *longitarsis* Kieffer grouped into the same cluster, while remaining specimens of *T*. *telmaticus*, *T*. *lestagei* and *Tanytarsus* cf. *dispar* Lindeberg could be well differentiated by DNA barcodes (Fig 11). However, there are currently several species with multiple synonyms within this group. Thus, perhaps Lindeberg’s [58] separation of sympatric species turns out to be closer to the true species boundaries within this aggregate than Ekrem’s [32] interpretation (and synonymization) of species in the same group.

**Fig 12:**
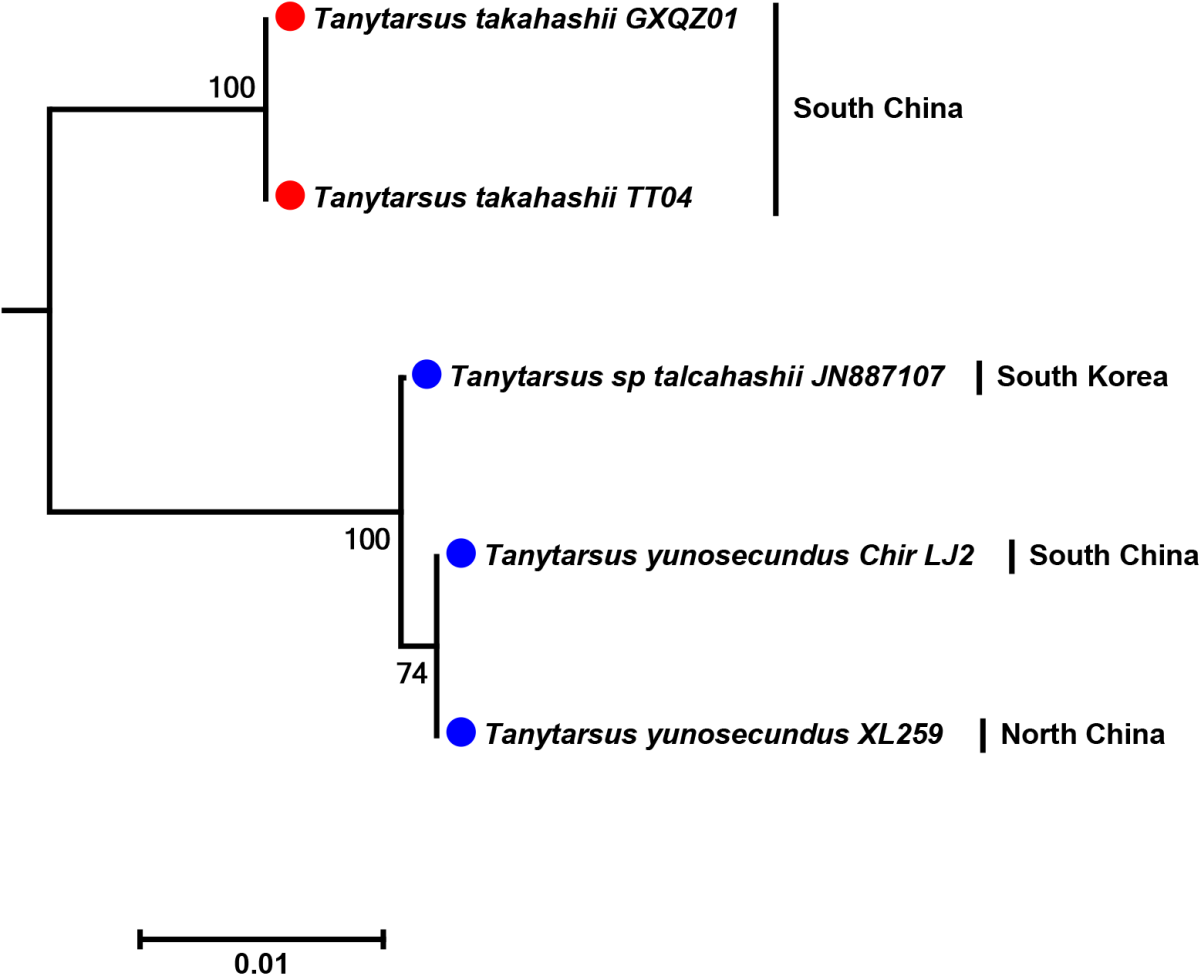
Neighbor joining subtree of Asian *Tanytarsus lestagei* aggregate (*Tanytarsus takahashii* and *Tanytarsus yunosecundus*). Numbers on branches represent bootstrap support (>70%) based on 500 replicates. It should be noted that “*Tanytarsus talcahashii*” is a misspelling of “*Tanytarsus takahashii*” in GenBank; scale represents K2P genetic distance.

The Asian members of the *T*. *lestagei* aggregate, *T*. *takahashii* and *Tanytarsus yunosecundus* Sasa previously have been distinguished from each other based on characters found in adult males, e.g. differences in the fore leg ratio and the shape of the superior volsella [32]. Recently, Tadashi Kobayashi (pers comm.) suggested *T*. *takahashii* to be a junior synonym of *T*. *yunosecundus*. DNA barcodes of populations from China and South Korea revealed low interspecific pairwise distance (2%) (Fig 12). However, a single DNA barcode of *T*. *takahashii* from Japan downloaded from GenBank did not group with these sequences and was more than 13% divergent based on K2P-distances. The single, divergent sequence was obtained from a pooled sample of male individuals (Richard Cornette pers comm.) and it is not unlikely that the barcode of *T*. *takahashii* from Japan in GenBank belong to another species. We have examined specimens from the same collection sample and can confirm that there are two superficially similar *Tanytarsus* and one *Cladotanytarsus* species present. Nevertheless, a synonymy of *T*. *takahashii* and *T*. *yunosecundus* should be avoided until more specimens of these species are examined and analyzed, especially from Japanese populations.

It should be kept in mind that there are no shortcuts to resolve the taxonomy of morphologically and genetically challenging species. Thus, further study using nuclear markers and more thorough morphological analyses are needed to sort out species boundaries and conclude on the potentially cryptic species within the different group treated above.

We based our study on taxon samples from around the world, particularly from Australia, China, Northern America and Western Europe. As there are more than 200 described *Tanytarsus* species from Africa, Australia, Eastern Asia and Southern America [32, 37], we have only about 1/3 of the known diversity of this genus. As shown above, a single or a few barcodes may not represent the putative species as whole, especially for geographically widespread species. Thus, restricted taxon sampling in many cases probably have led to an underrepresentation of the complete genetic range and inaccuracies in estimation of species [89]. As mentioned above, geographically separated populations of *T*. *occultus* showed high intraspecific variability. Without morphological taxonomic consideration, the two geographically separated populations would be regarded as cryptic species. In this and other sister group cases in *Tanytarsus*, a more detailed analysis is required to determine the current rate of gene flow between populations and if there is speciation in progress. Despite several challenging and biologically interesting incidents, DNA barcoding generally is effective for species identification in *Tanytarsus*, even when taxa are sampled from multiple and large geographic areas. This is similar to what has been recorded for Lepidoptera [90, 91] but opposite to findings for aquatic beetles [92]. Our data and results also show that traditional taxonomic considerations and comprehensive sampling are highly important for correct identification [87, 93] and that DNA barcodes in reference libraries provide an excellent starting point for taxonomic considerations and discussion on the identity of challenging taxa.

### OTU estimation

Any particularly set threshold value for species separation will affect taxon diversity in any taxonomic group. Moreover, studies indicate that the same threshold is not appropriate for all groups. In insects for instance, a 2% threshold provides effective identification at the species-level of Ephemeroptera [94–96], Lepidoptera [97, 98], Plecoptera and Trichoptera [97]. While a 2.2% threshold has been found appropriate for Heteroptera [99, 100], a 2.5% threshold has been found suitable for aquatic beetles [101], a >3% threshold has been registered for several dipteran groups [46, 102, 103].

In Chironomidae, average intraspecific divergences range from 0.9% to 2.32% [3, 104] and when disregarding obvious cryptic species clusters, maximum intraspecific K2P distances can be as high as 8.5%, considerably higher than comparable rates in Heteroptera, Hymenoptera and Lepidoptera. In our study, morphologically determined *Tanytarsus* species had average intraspecific divergences of 2.14% (S2 Table). This was the case even for heavily sampled species such as *T*. *mendax*, where 430 sequences showed a mean pairwise divergence of 2.1%.

The number of OTUs in a DNA barcode dataset relies on both the method and threshold value used. Thus, we tested different methods for OTU calculation to explore what might be an appropriate threshold for *Tanytarsus* species.

**Fig 13:**
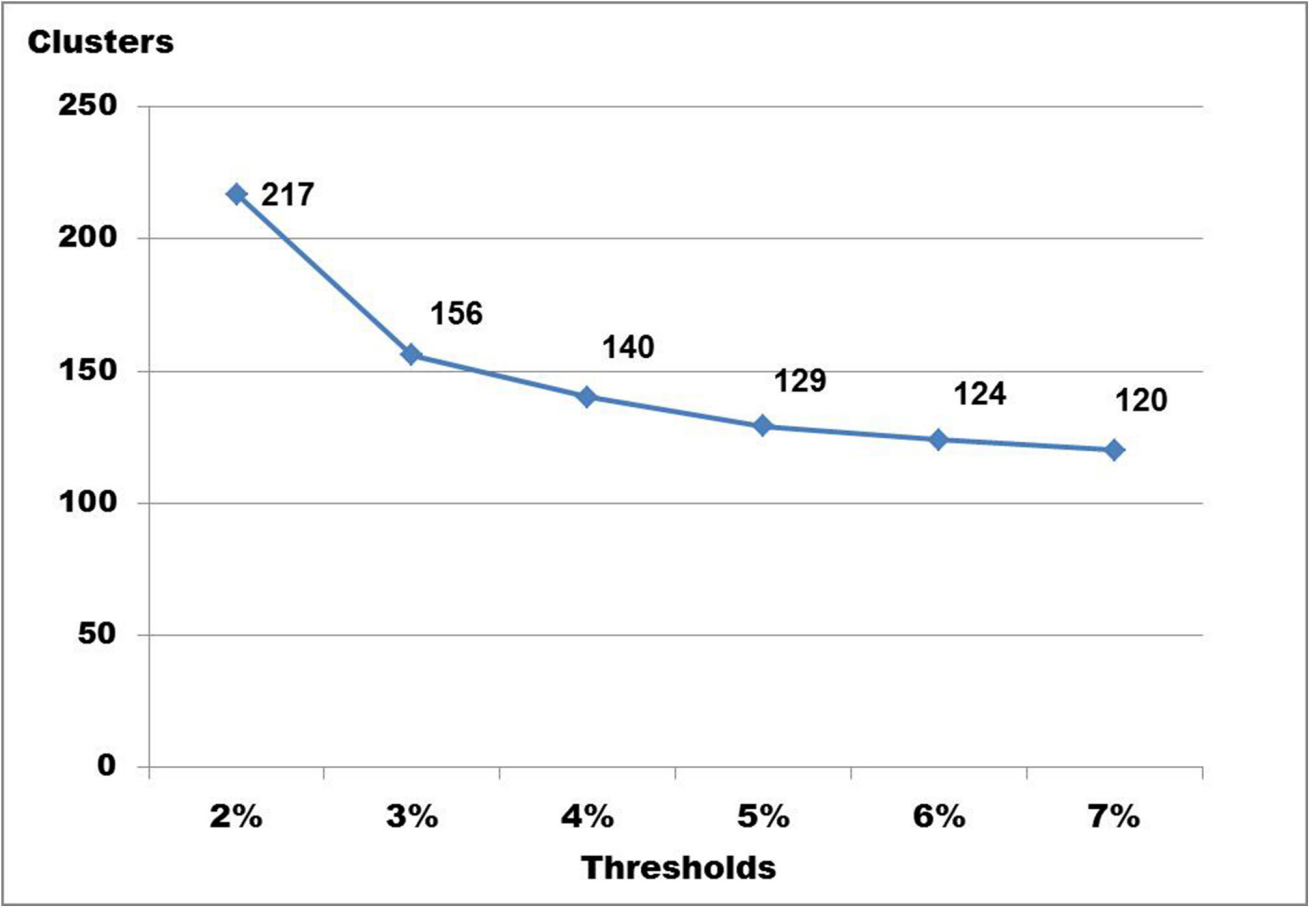
The number of DNA clusters according to Objective Clustering at different thresholds.

**Fig 14:**
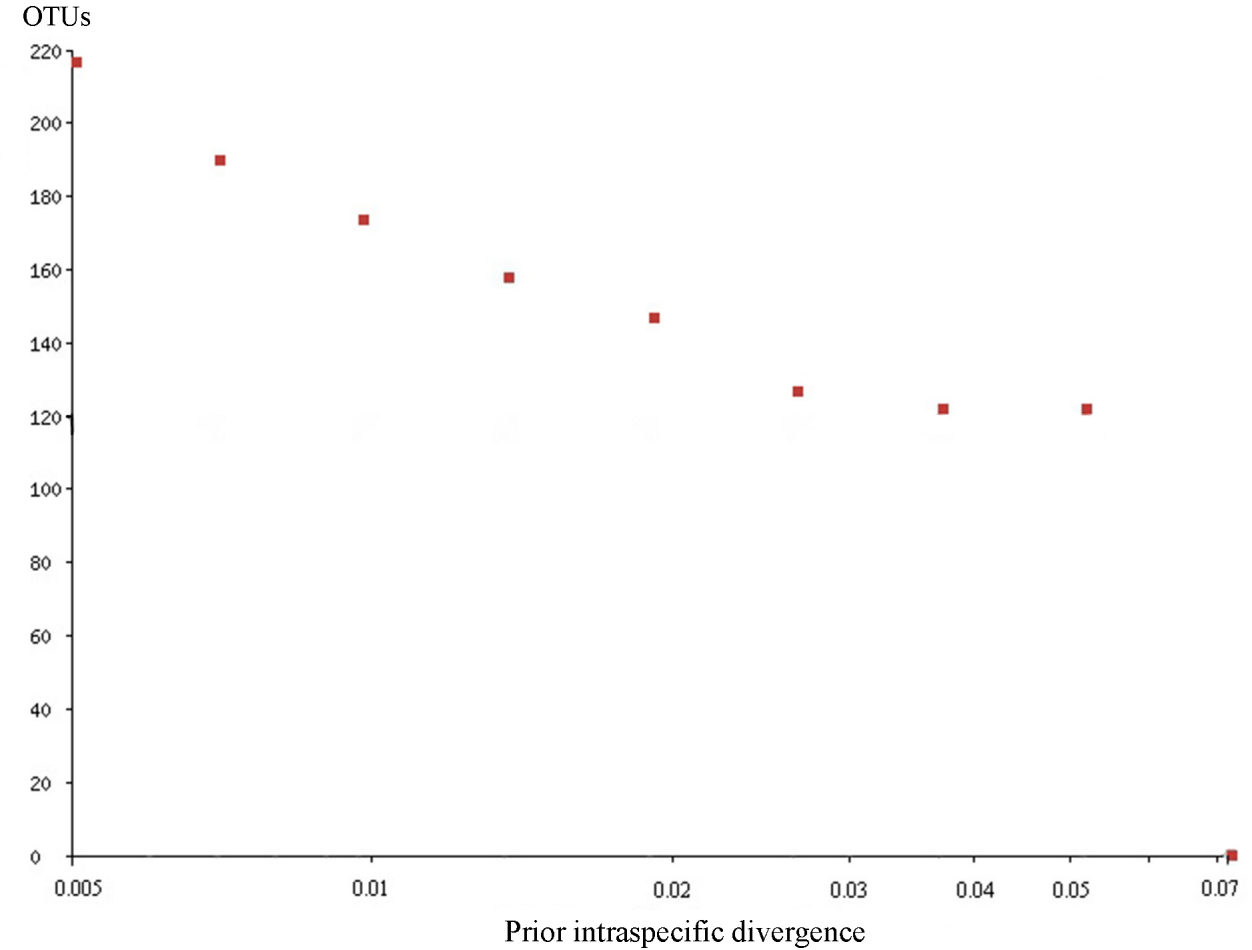
The number of the OTUs by the prior intraspecific divergence calculated with ABGD online.

**Fig 15:**
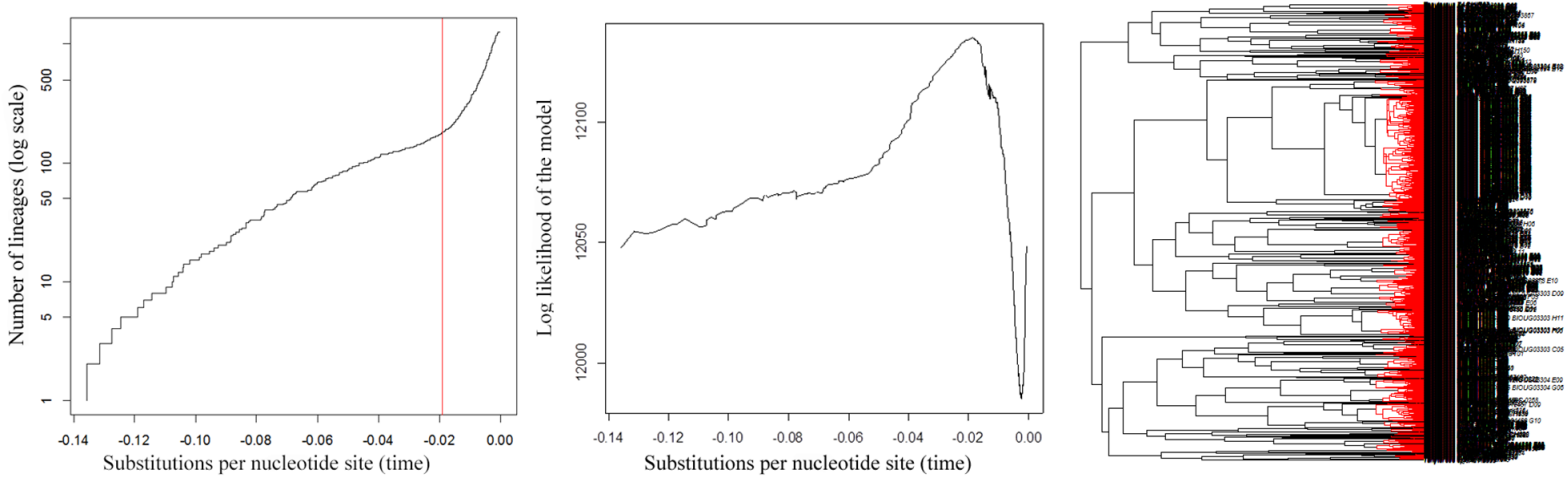
Results of the GMYC analysis. The red vertical line (left) indicates the single threshold time between inter-intraspecific branching; y axis (left) equals the number of lineages; y axis (center) equals the log likelihood of the single threshold GMYC model; the x axes (left and center) show substitutions per nucleotide site; the red branches (right) on the gene tree indicate estimated delimited species.

Using Objective Clustering at threshold 2% yielded 217 clusters, while thresholds ranging from 3% to 7% yielded 120–156 clusters (Fig 13). Applying the ABGD method with prior intraspecific divergence ranging from 3%–5% yielded 123–129 OTUs (Fig 14). This is similar to the number of divergent barcode clusters seen when subjectively evaluating the neighbor joining tree. Analyses of the reduced dataset containing only unique haplotype sequences yielded 180 clusters with a confidence interval ranging from 164–193 using GMYC (Fig 15) and 224–225 clusters with PTP and bPTP (S3 File). Thus, GMYC yielded a more conservative number of species than PTP, but still considerably higher than what was obtained with Objective Clustering and ABGD using higher thresholds and our subjective evaluation of the neighbor joining tree. The observed difference in the species estimate for PTP may be associated with the unbalanced number of individuals sampled per species as this can affect the species delimitation [45].

Examining the dataset in BOLD, 2749 of 2790 barcodes were assigned a barcode index number and represented 242 BINs. In total, 2250 barcodes matched with morphospecies, representing 166 BINs, and 63 barcodes were singletons representing 63 BINs. Surprisingly, the 242 BINs in BOLD were approximately twice the number of divergent barcode clusters observed in the neighbor joining tree (131) and even higher than the number of species estimated by PTP. Thus, for *Tanytarsus*, the number of BINs generated by the BIN algorithm did not represent morphological species well. One reason might be that numerous identical haplotypes move the BIN-boundary upwards (Sujeevan Ratnasingham pers comm.). However, since BINs in BOLD have been shown to coincide strongly with known species boundaries in other insect groups, i.e. Coleoptera and Lepidoptera [97, 105], we suspect that it also is the starting threshold for the BIN algorithm that is too low for Chironomidae. Comparison of the results of ABGD, GMYC, PTP and objective clustering indicate that a 4–5% threshold is more appropriate for species delimitation in genus *Tanytarsus*.

## Conclusion and Future Prospects

The discrimination of *Tanytarsus* species by DNA barcodes was highly successful with unambiguous grouping of 94.6% of the species recognized through prior morphological study. Deep intraspecific divergence existed in some species complexes, and further taxonomic studies are required to resolve these issues. Such studies preferably should involve morphological examination of all life stages as well as analysis of relationships using nuclear markers. Morphological re-examination of voucher specimens, in particular nominal types will be crucial to sort out taxonomic challenges and provide the best barcode reference library possible. We suggest that a 4–5% threshold on average is an appropriate level for species separation in *Tanytarsus* non-biting midges. This threshold is considerably higher than it is for certain other insect groups as well as the basis for the BIN-algorithm used in BOLD.

## Supporting Information

S1 File Neighbor joining bootstrap consensus tree for 2790 *Tanytarsus* barcodes. Numbers on branches are bootstrap support (>70%) using 500 bootstrap replicates. The clade names in blue represent 28 groups morphologically unidentified to the species-level, but clustering together. The clade names in red represent 16 identified morphospecies which likely are new to science but unpublished. The clade names in black represent morphospecies. For named species with more than two clusters, indicating cryptic species or misidentifications, we have used symbols with the same color and shape in front of the sequence names.
(PDF)

S2 File Original alignment of 2790 *Tanytarsus* barcode sequences. Alignment of the 2790 sequences based on the Muscle algorithm (Edgar 2004) in MEGA6. Final length 658 bp.
(FAS)

S3 File Maximum likelihood tree based on the PTP model. (PDF)

S1 Table Estimates of evolutionary divergence between sequences. Pairwise distance between 2790 nucleotide sequences based on the K2P model calculated in MEGA6. The analysis included all codon positions and pairwise deletion of gaps for each sequence pair.
(XLS)

S2 Table Estimates of average evolutionary divergence over sequence pairs within groups. Average pairwise distances within species based on the K2P substitution model calculated in MEGA6. The analysis included all codon positions and pairwise deletion of gaps for each sequence pair. In order to calculate the intra- and interspecific distances including sequences without species names in public databases, species names were added to 1242 DNA barcodes if they matched named sequences in the neighbor joining tree.
(XLS)

S3 Table Estimates of evolutionary divergence over sequence pairs between groups. Average pairwise distance between species based on the K2P substitution model calculated in MEGA6. The analysis included all codon positions and pairwise deletion of gaps for each sequence pair. In order to calculate the intra- and interspecific distances including sequences without species names in public databases, species names were added to 1242 DNA barcodes if the matched named sequence in the neighbor joining tree.
(XLS)
