# Supplementary figures and images for: Exploring Genetic Divergence in a Species-Rich Insect Genus Using 2790 DNA Barcodes
Source: PLoS One. 2015 Sep 25;10(9):e0138993. doi: 10.1371/journal.pone.0138993 (PMC4583400; doi:10.1371/journal.pone.0138993)

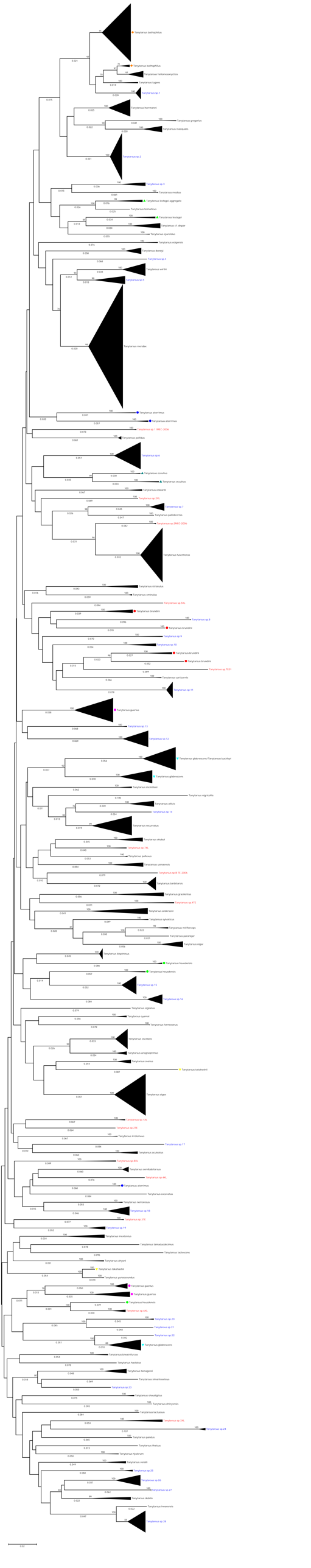

Supplement: S1 File — Numbers on branches are bootstrap support (>70%) using 500 bootstrap replicates. The clade names in blue represent 28 groups morphologically unidentified to the species-level, but clustering together. The clade names in red represent 16 identified morphospecies which likely are new to science but unpublished. The clade names in black represent morphospecies. For named species with more than two clusters, indicating cryptic species or misidentifications, we have used symbols with the same color and shape in front of the sequence names. (PDF) [file pone.0138993.s001.pdf]

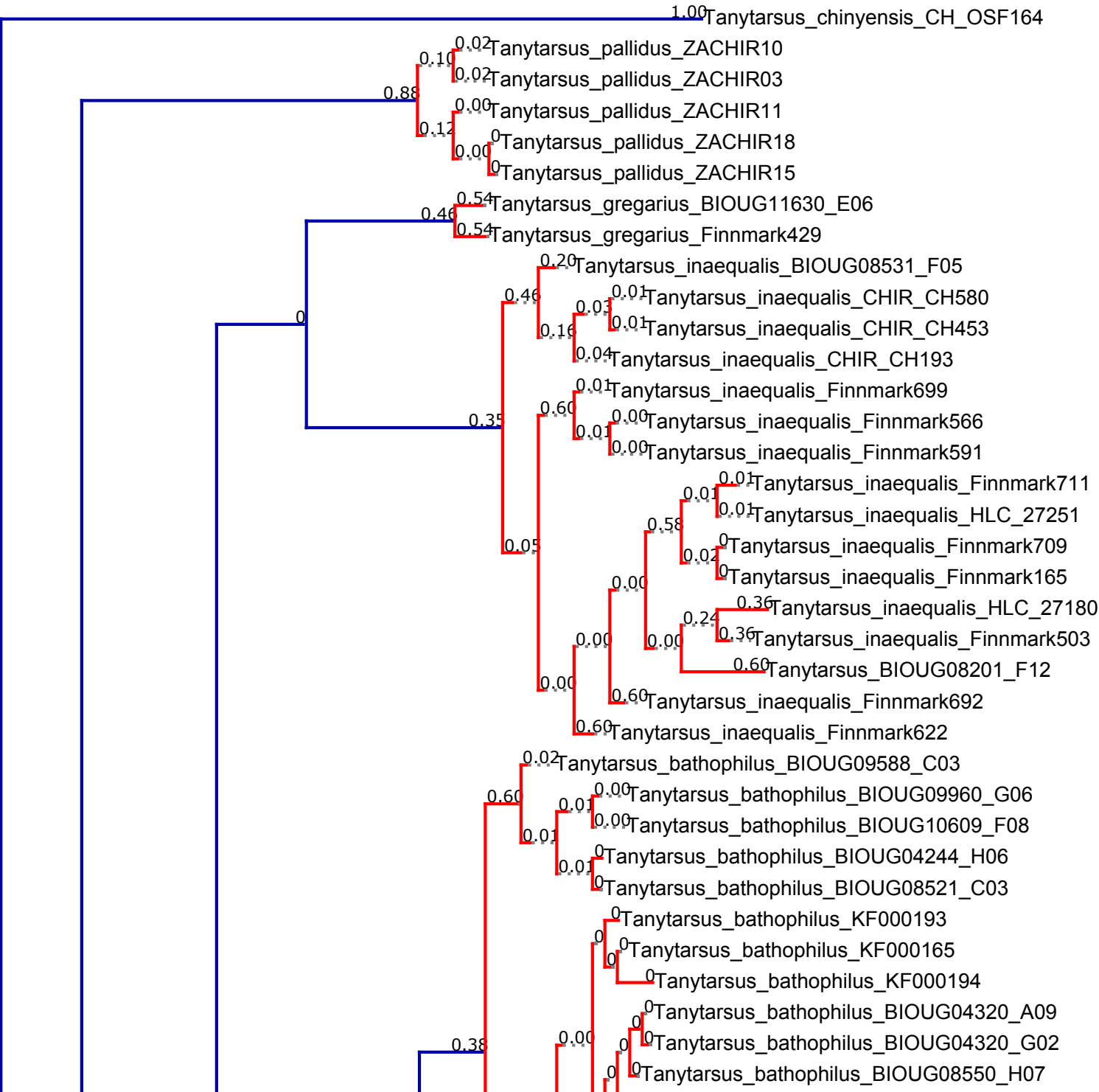

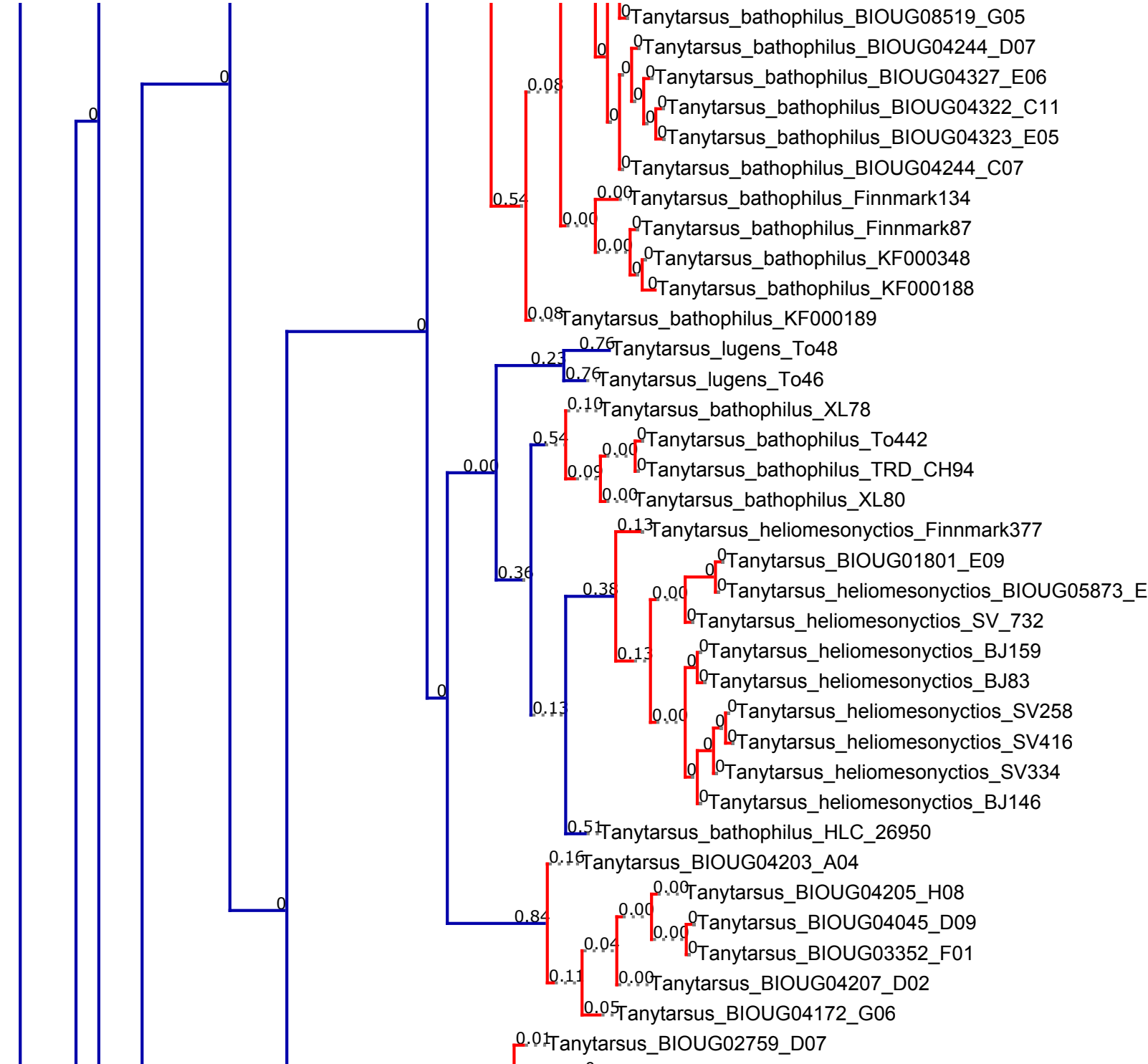

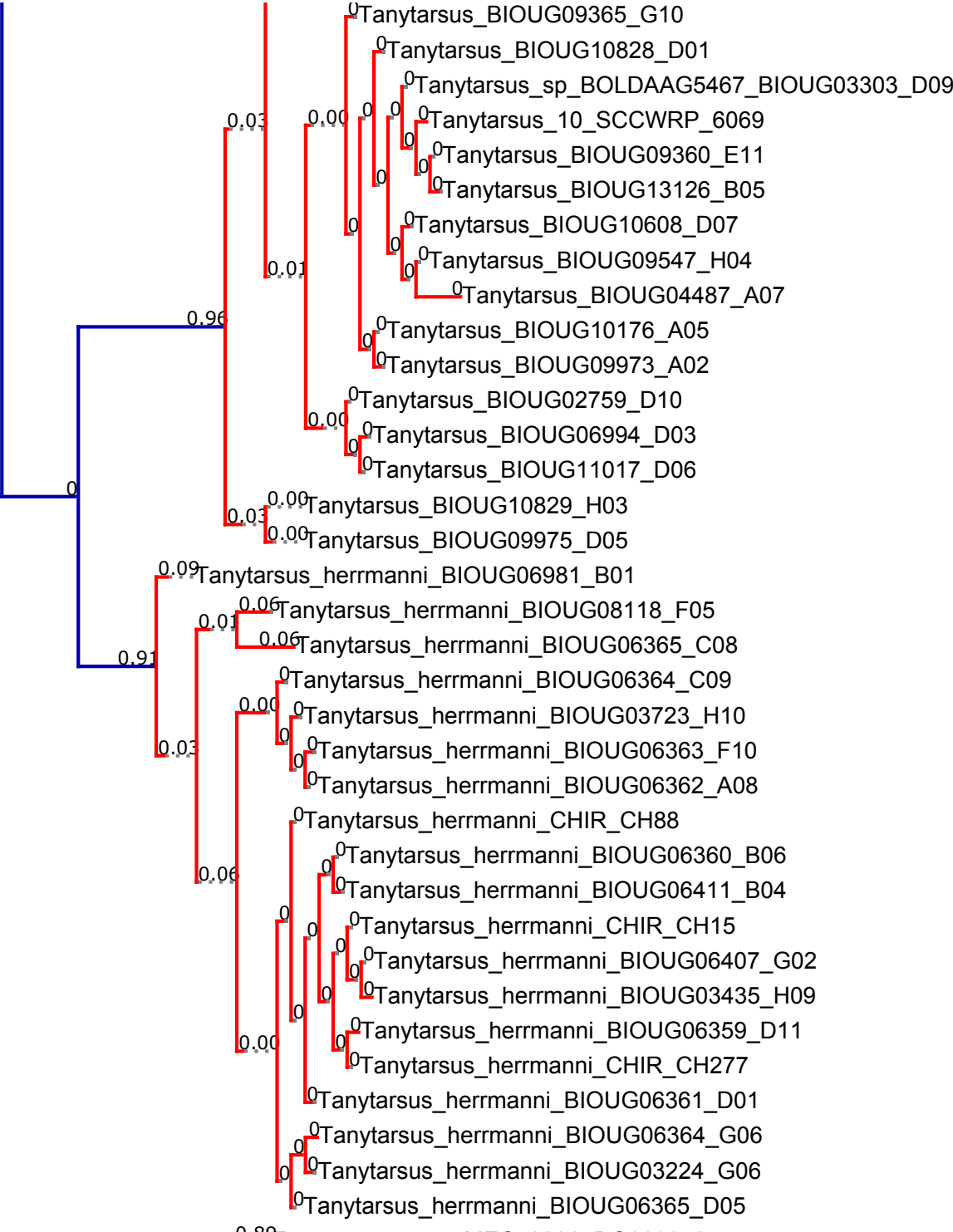

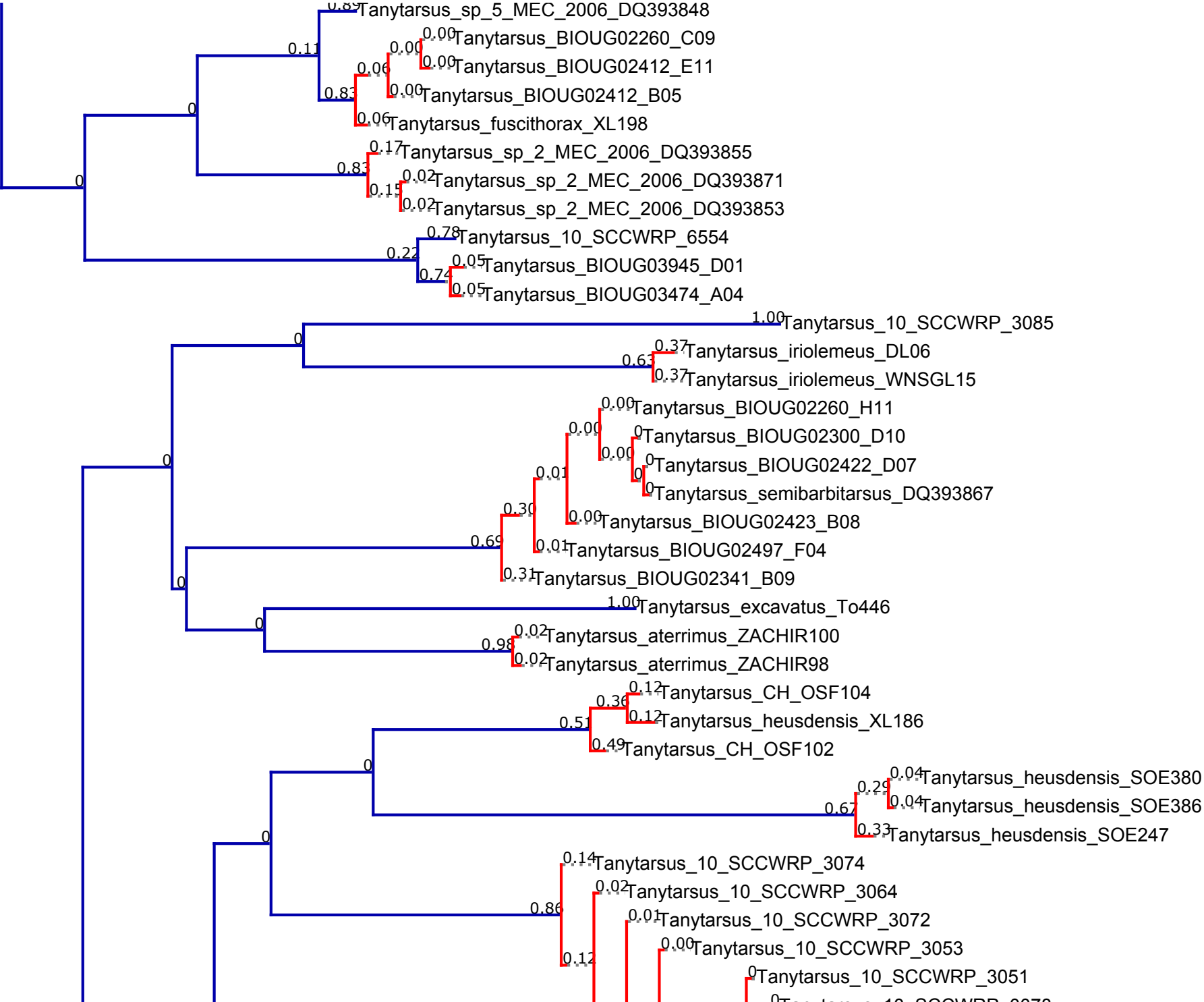

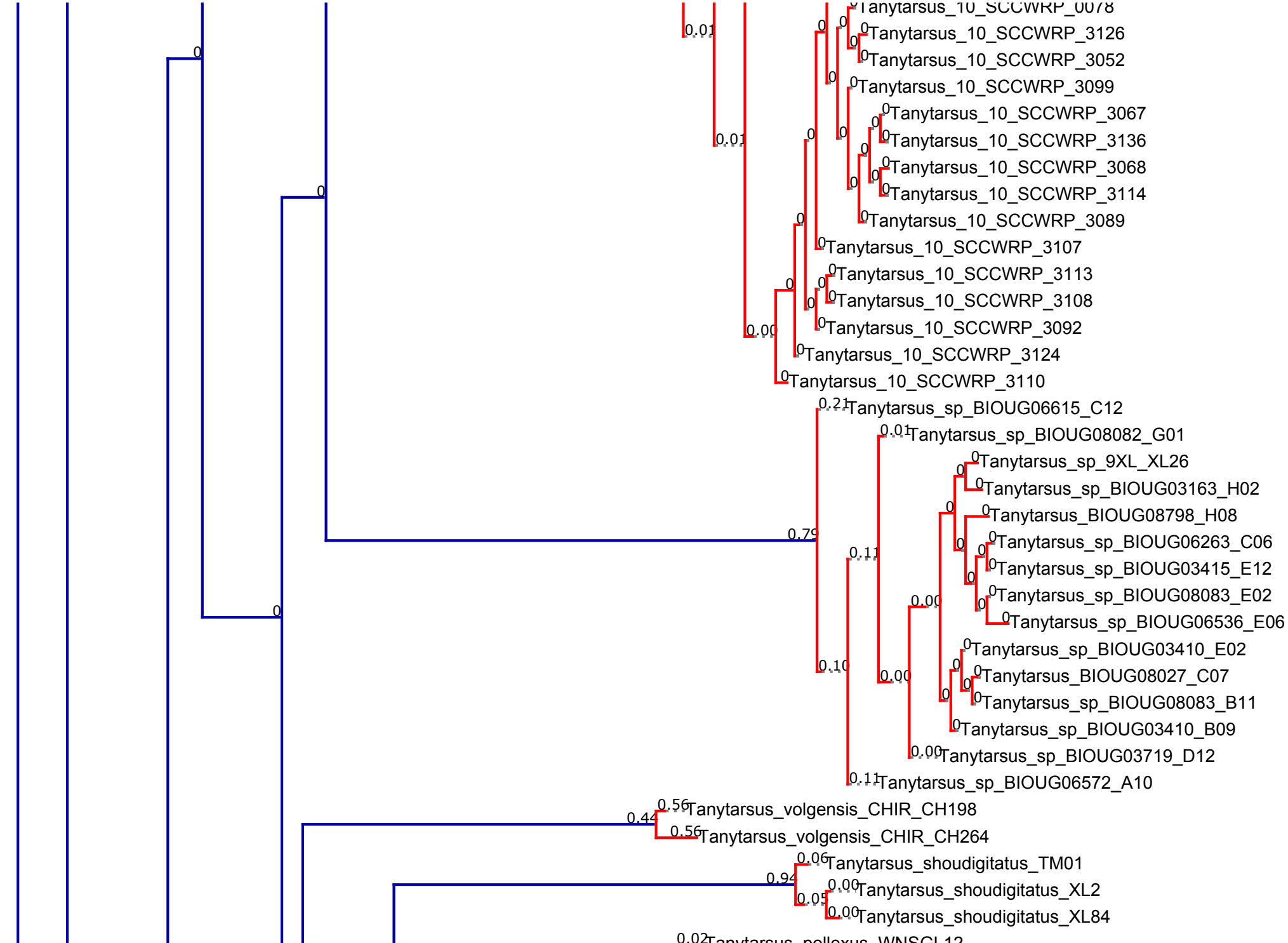

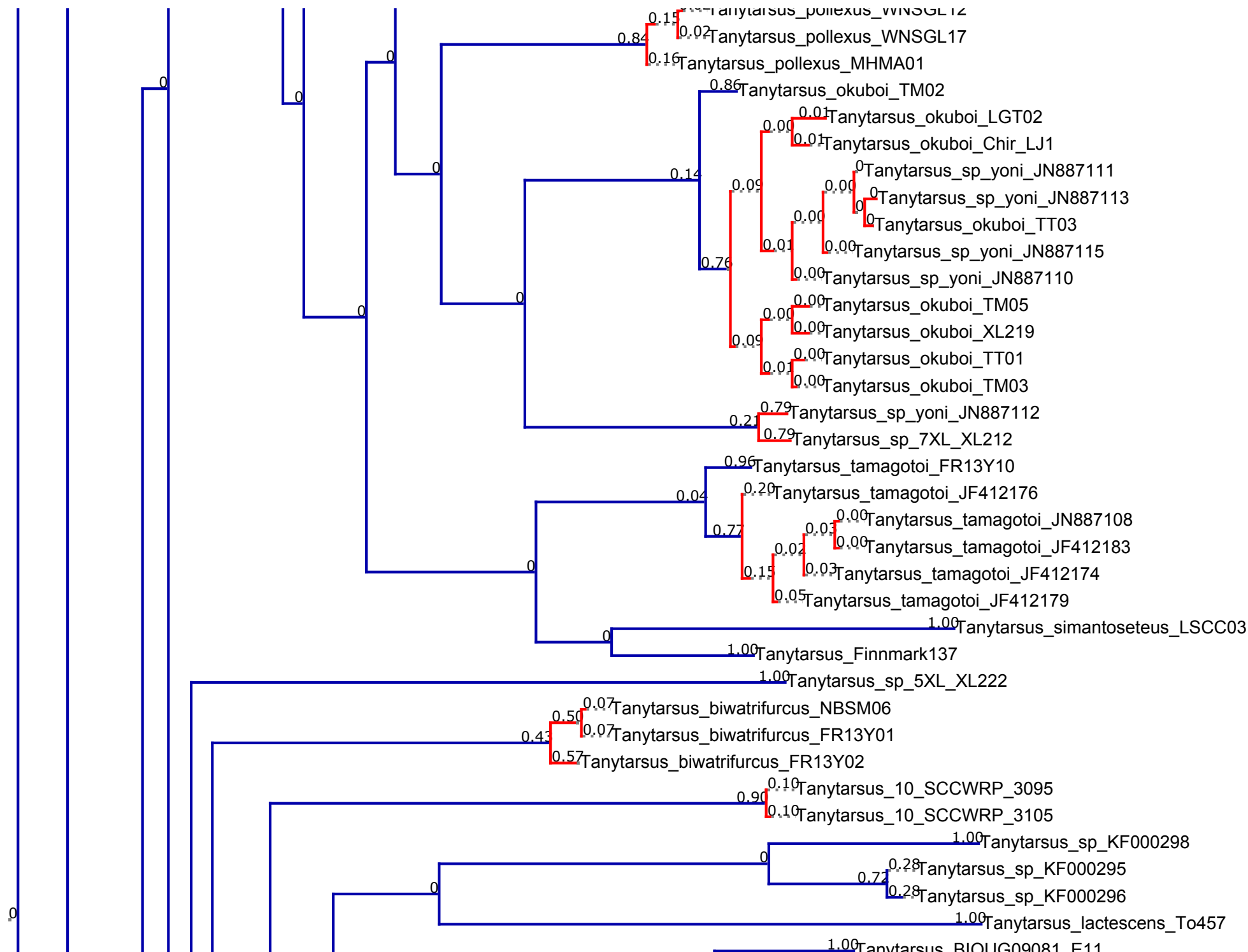

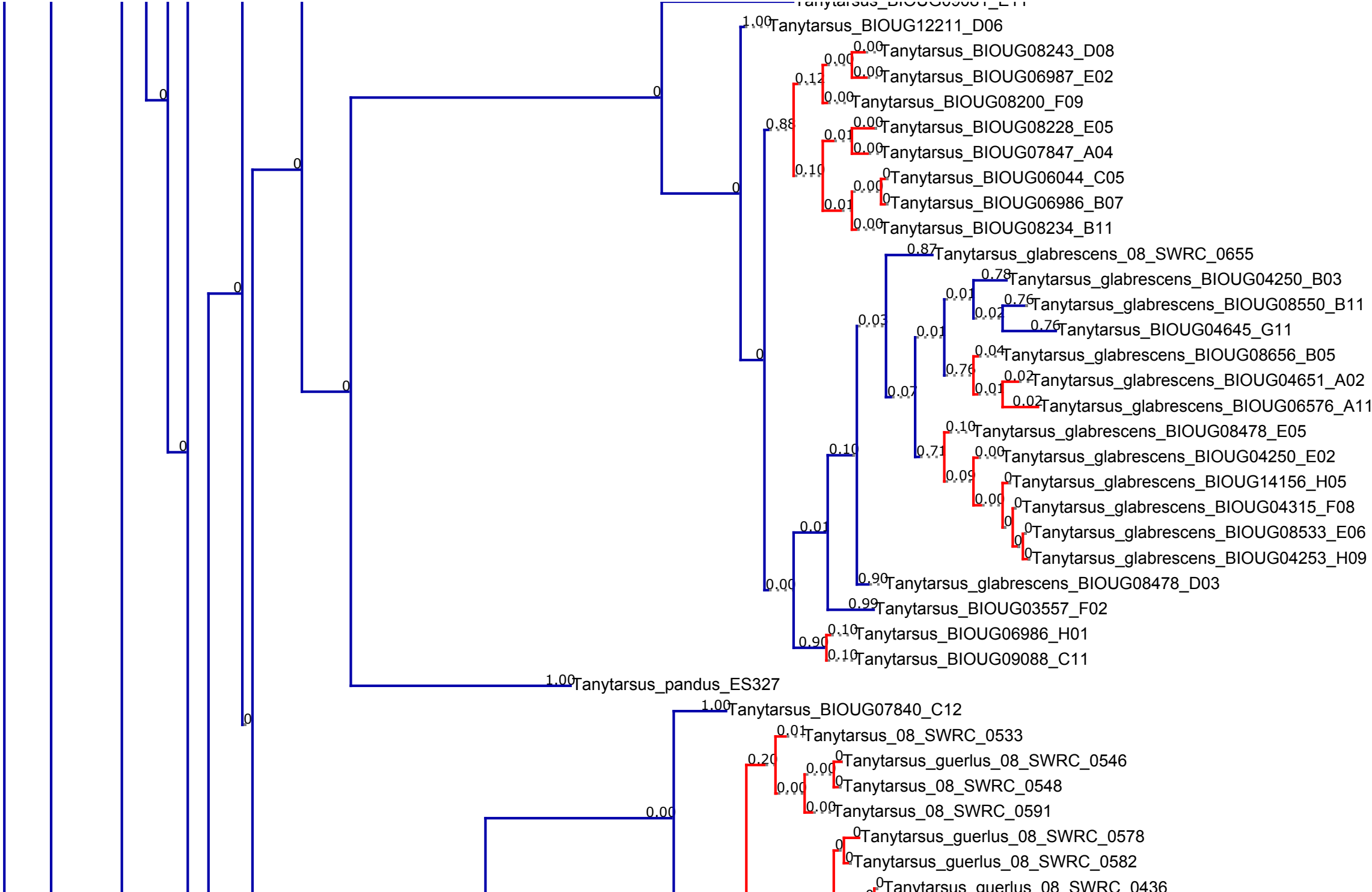

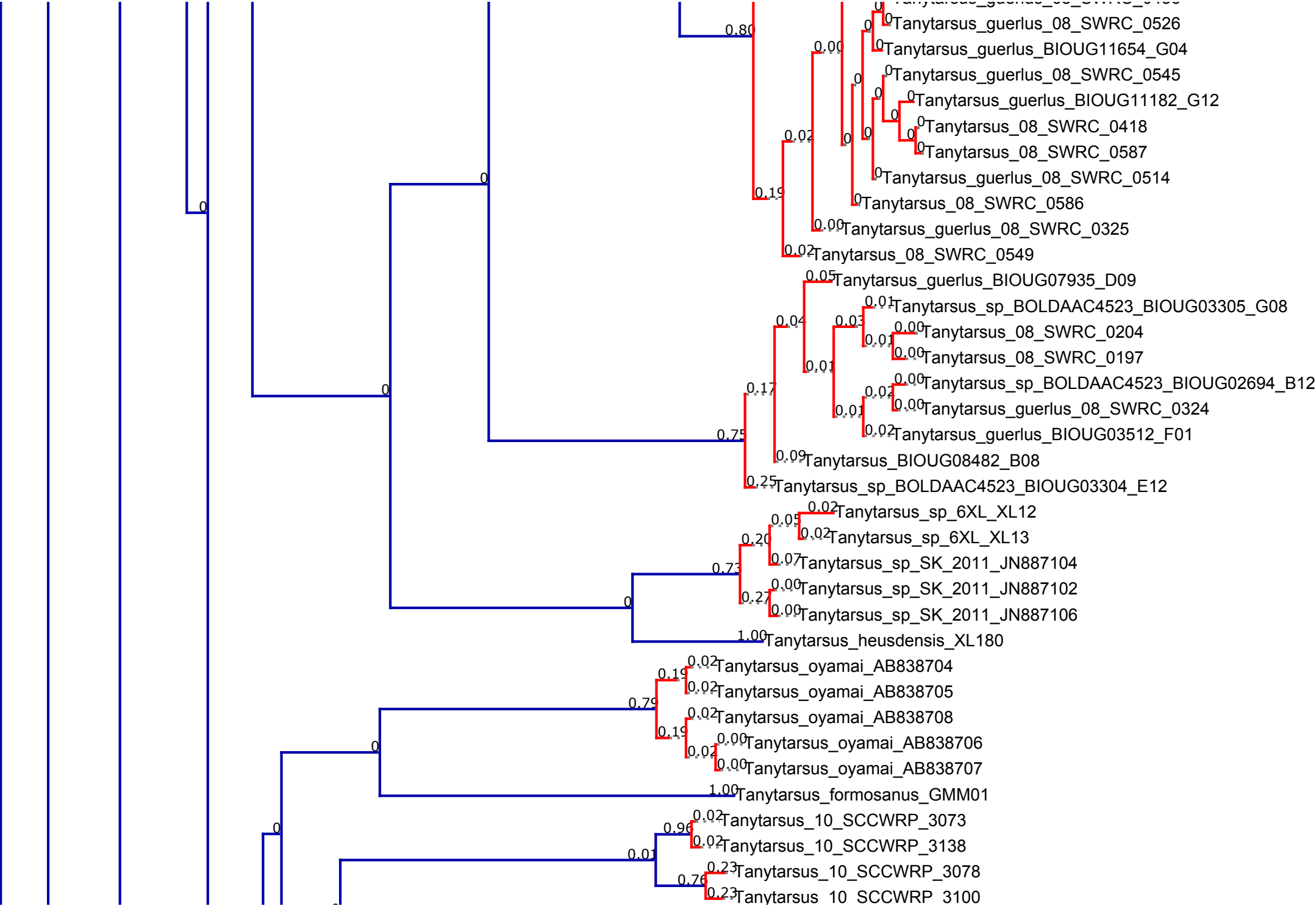

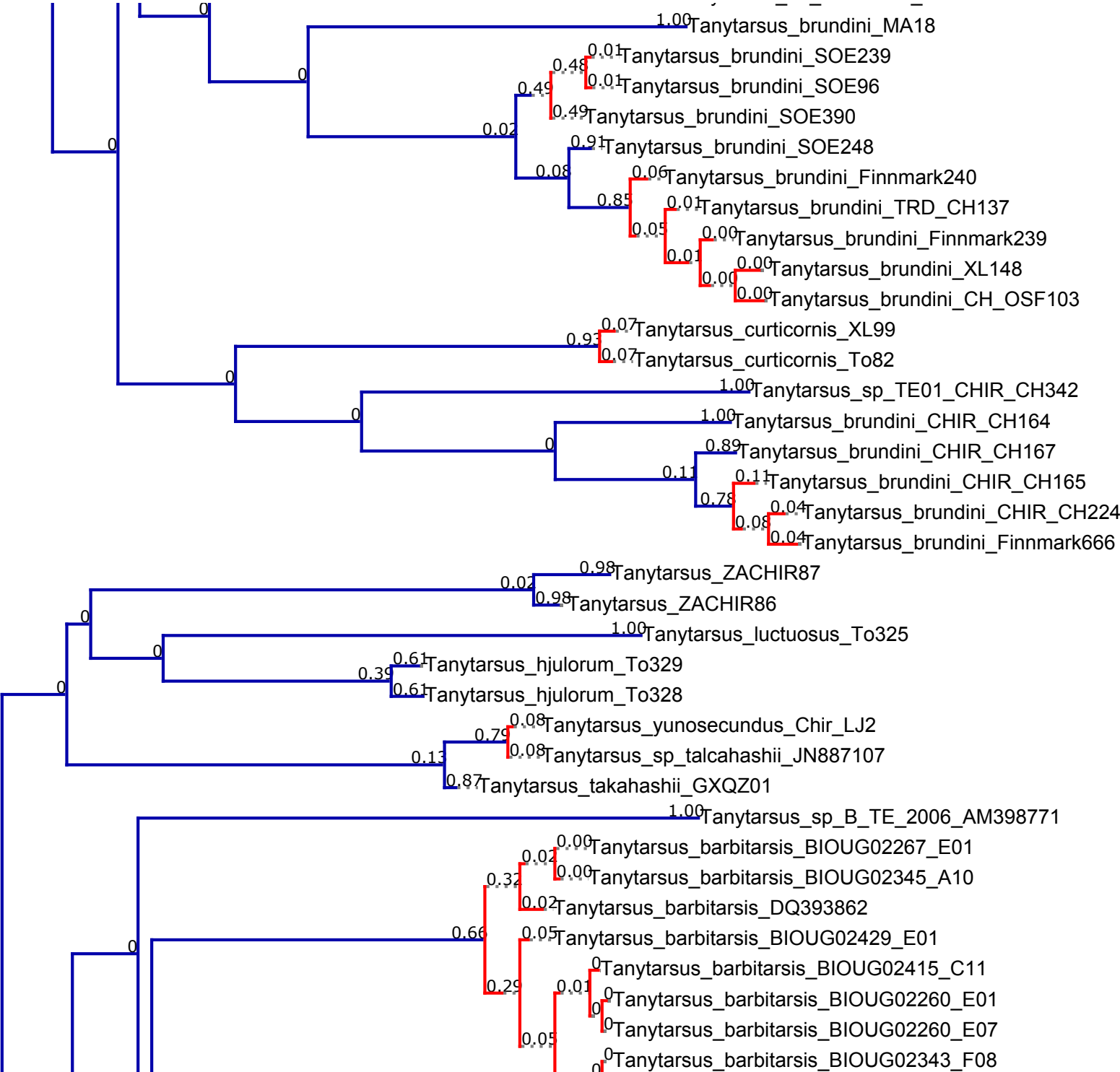

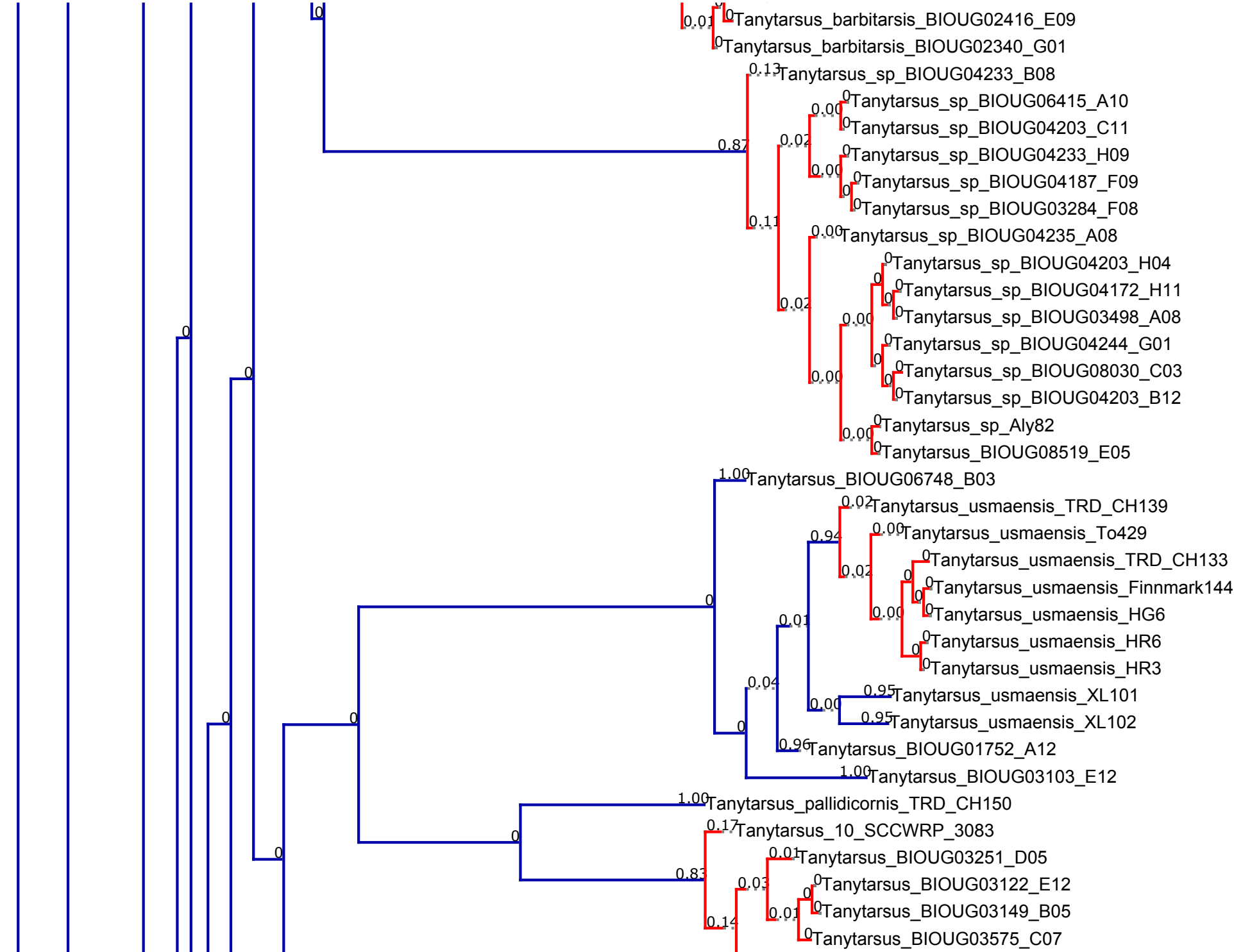

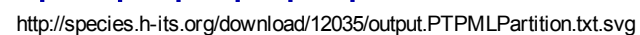

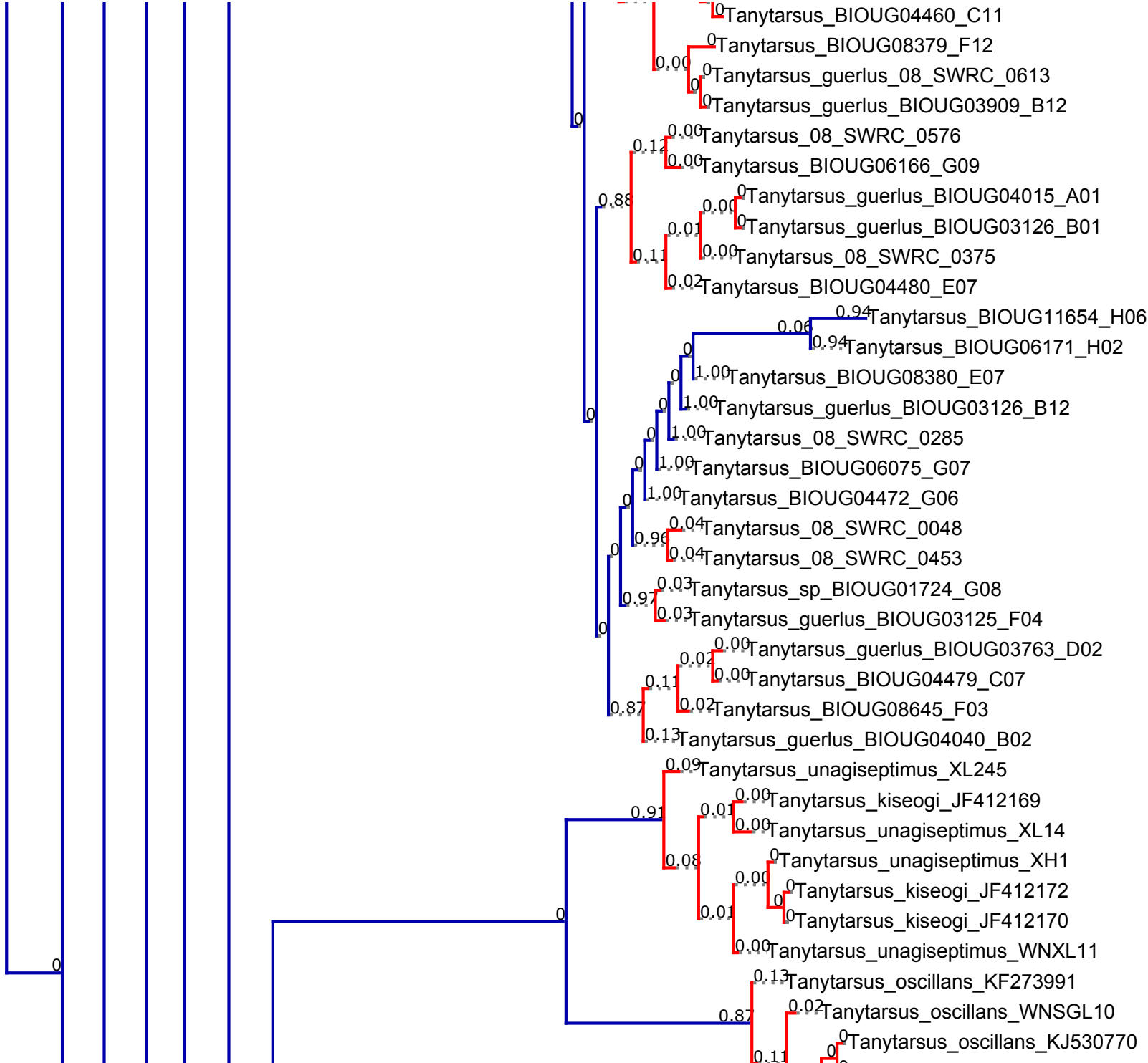

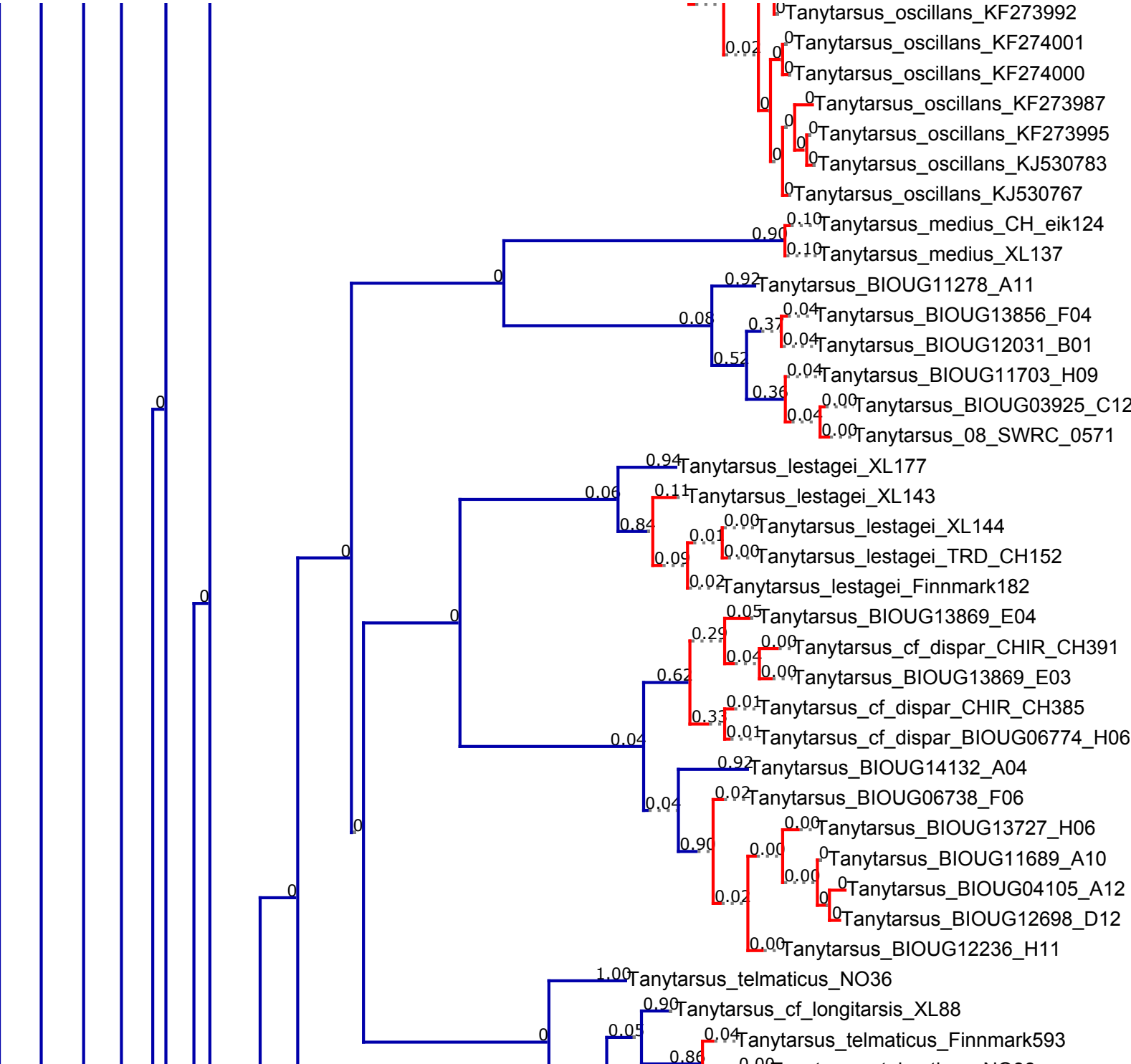

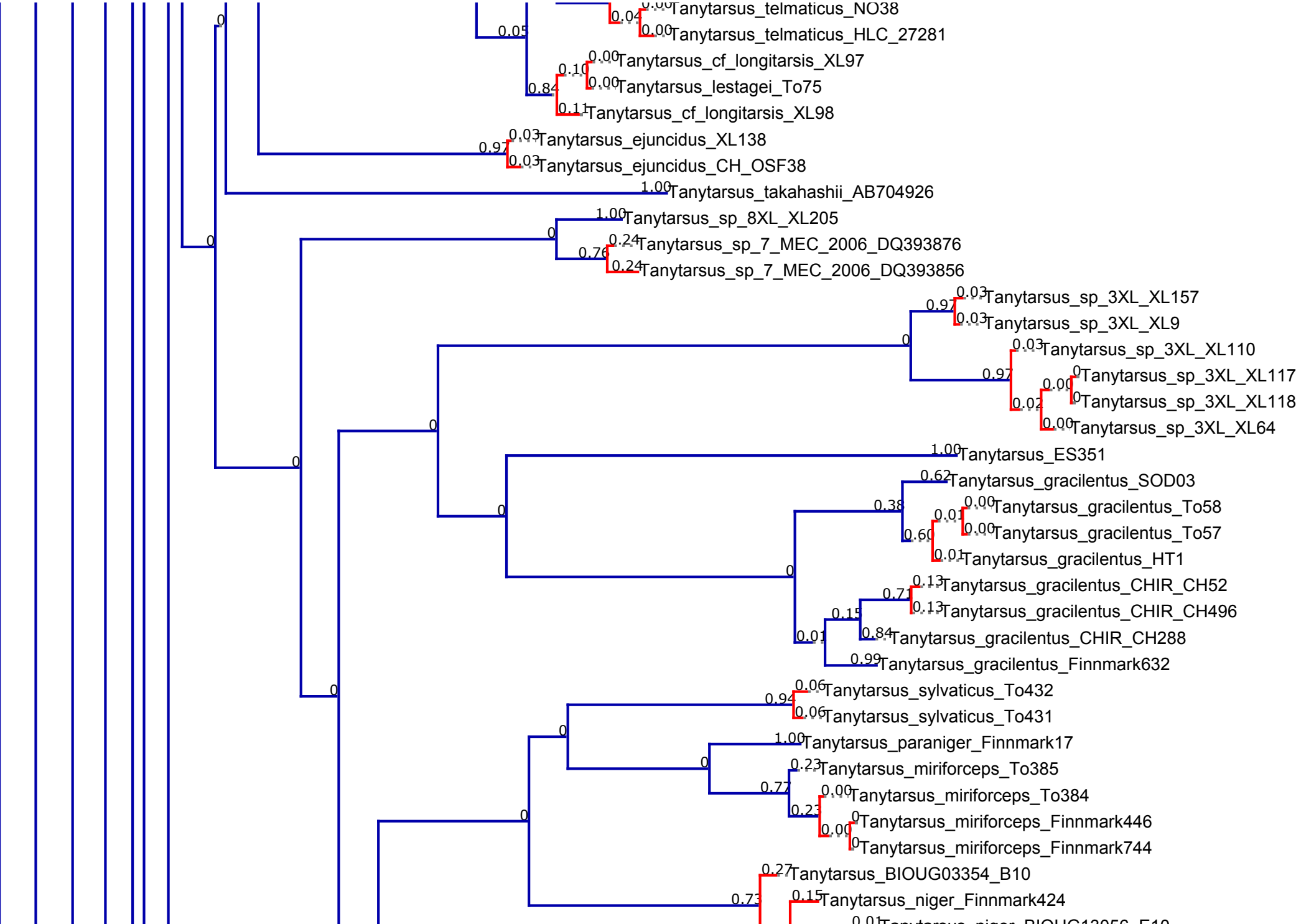

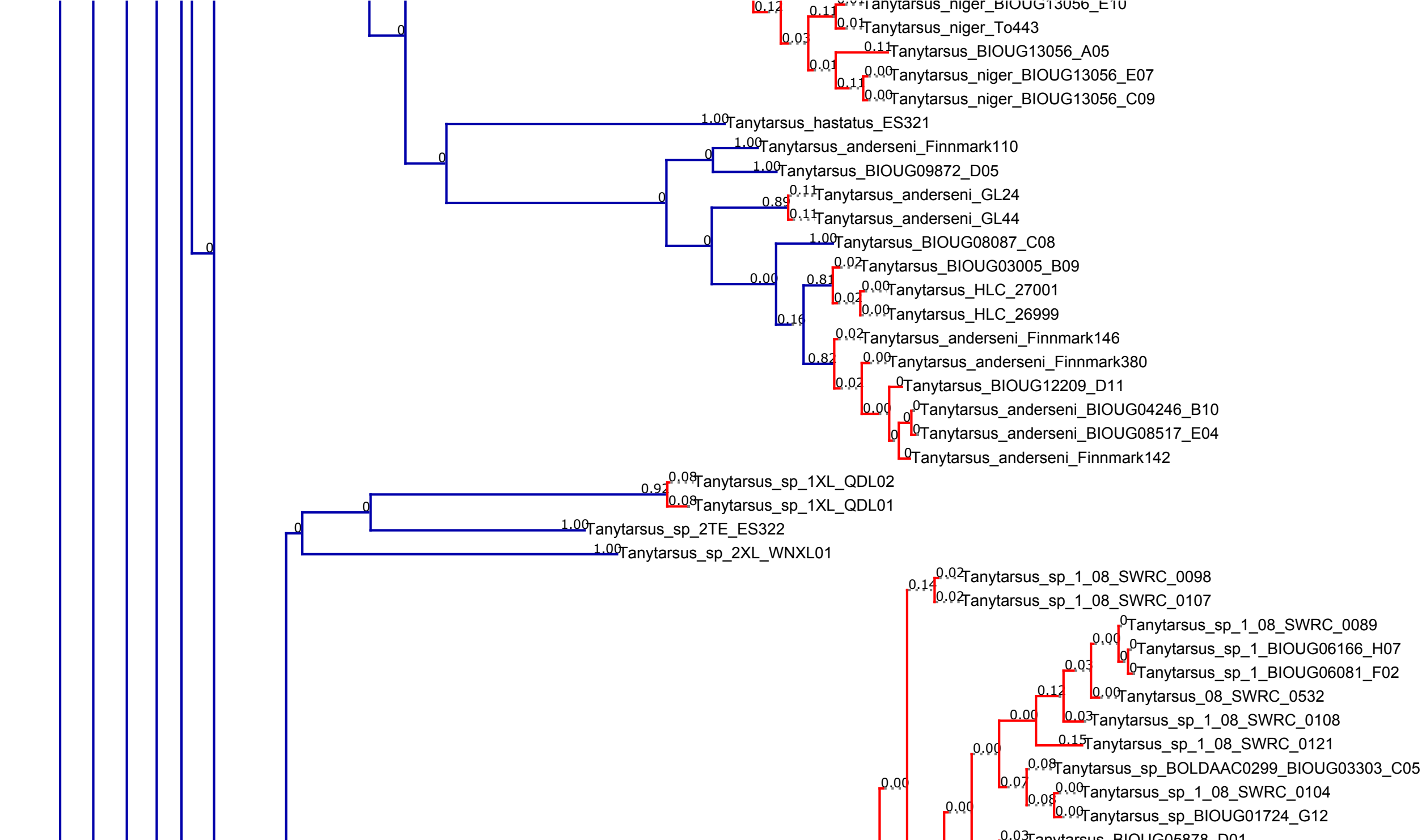

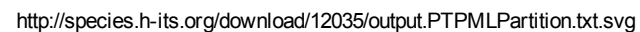

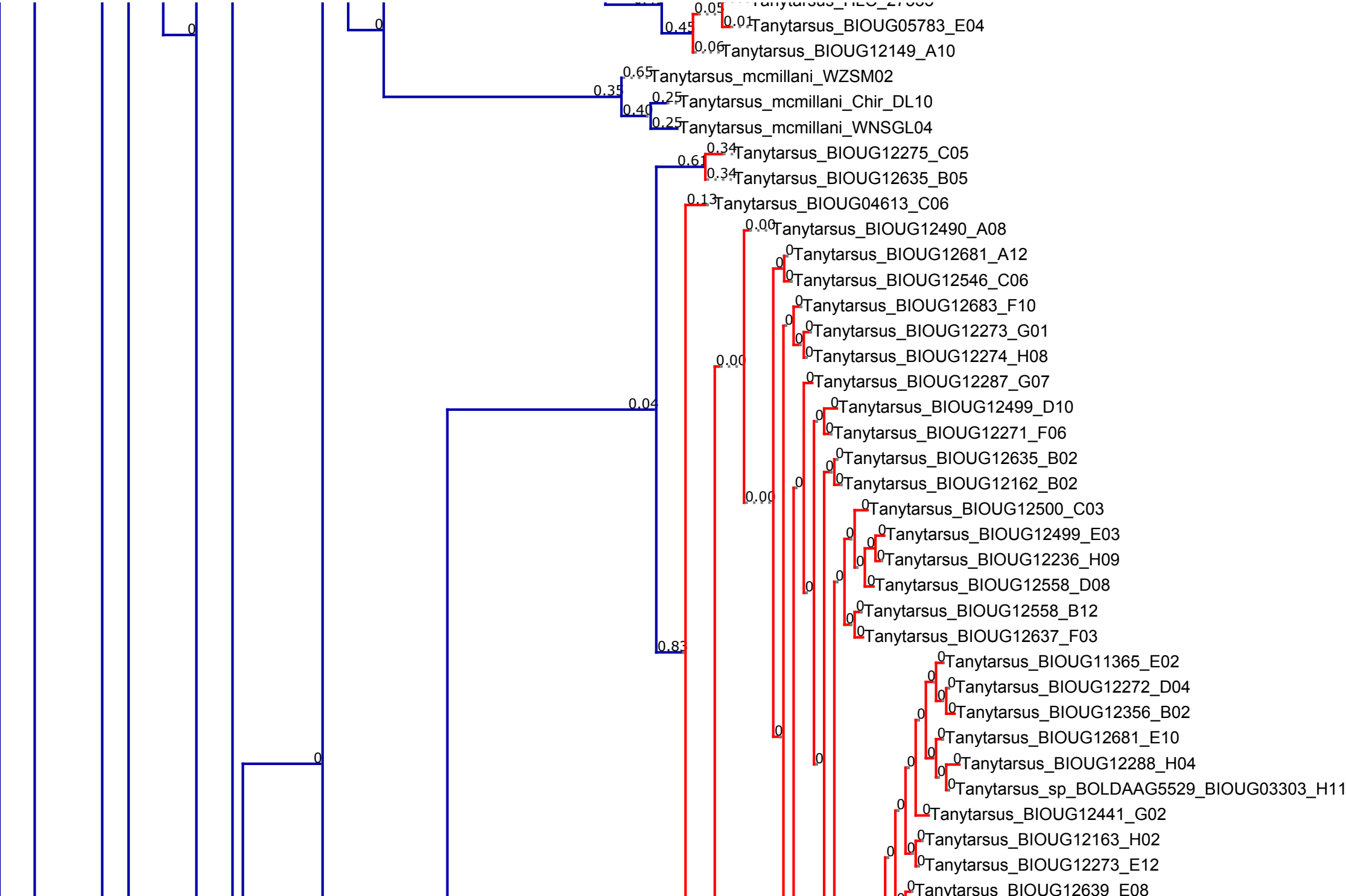

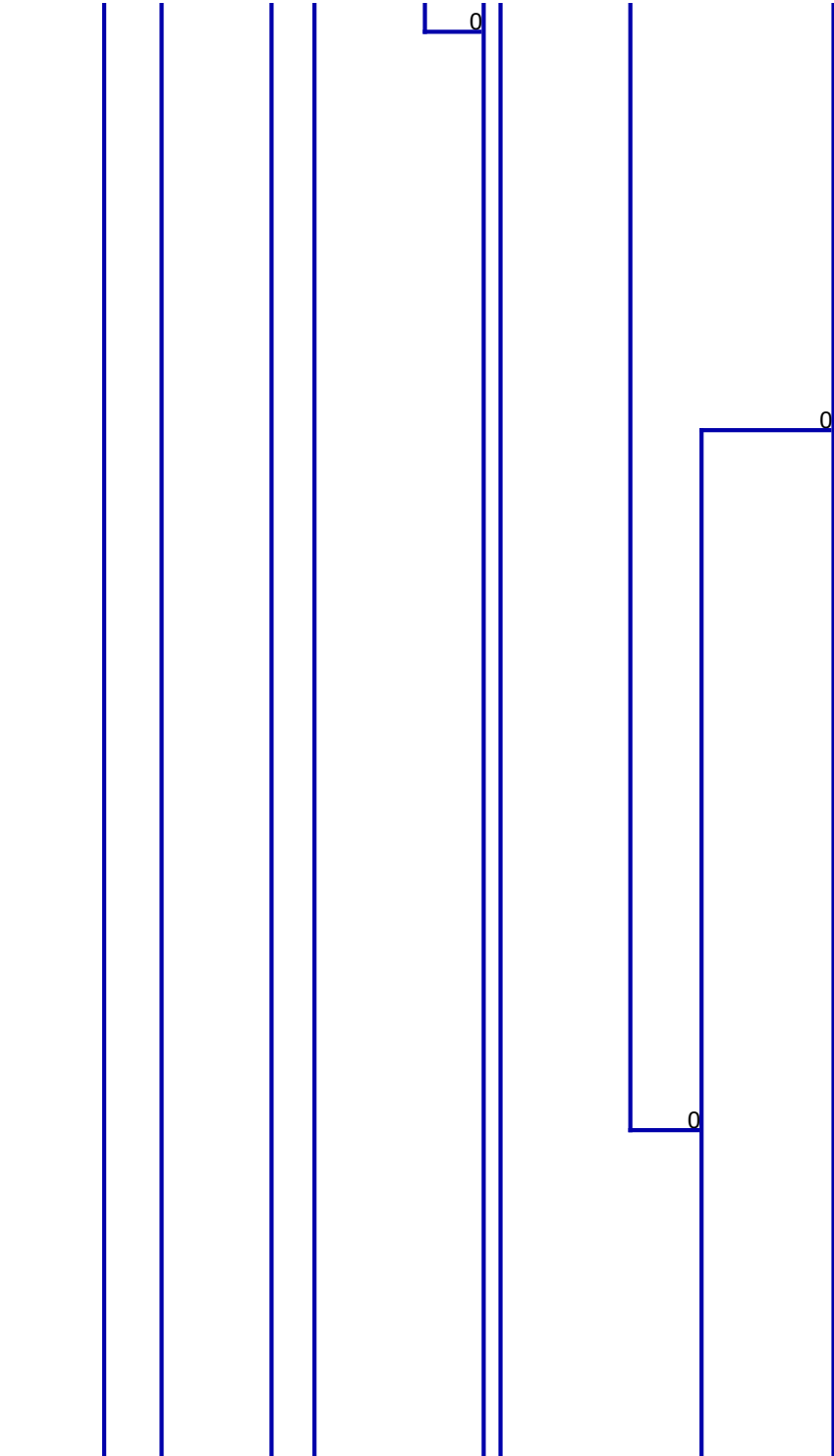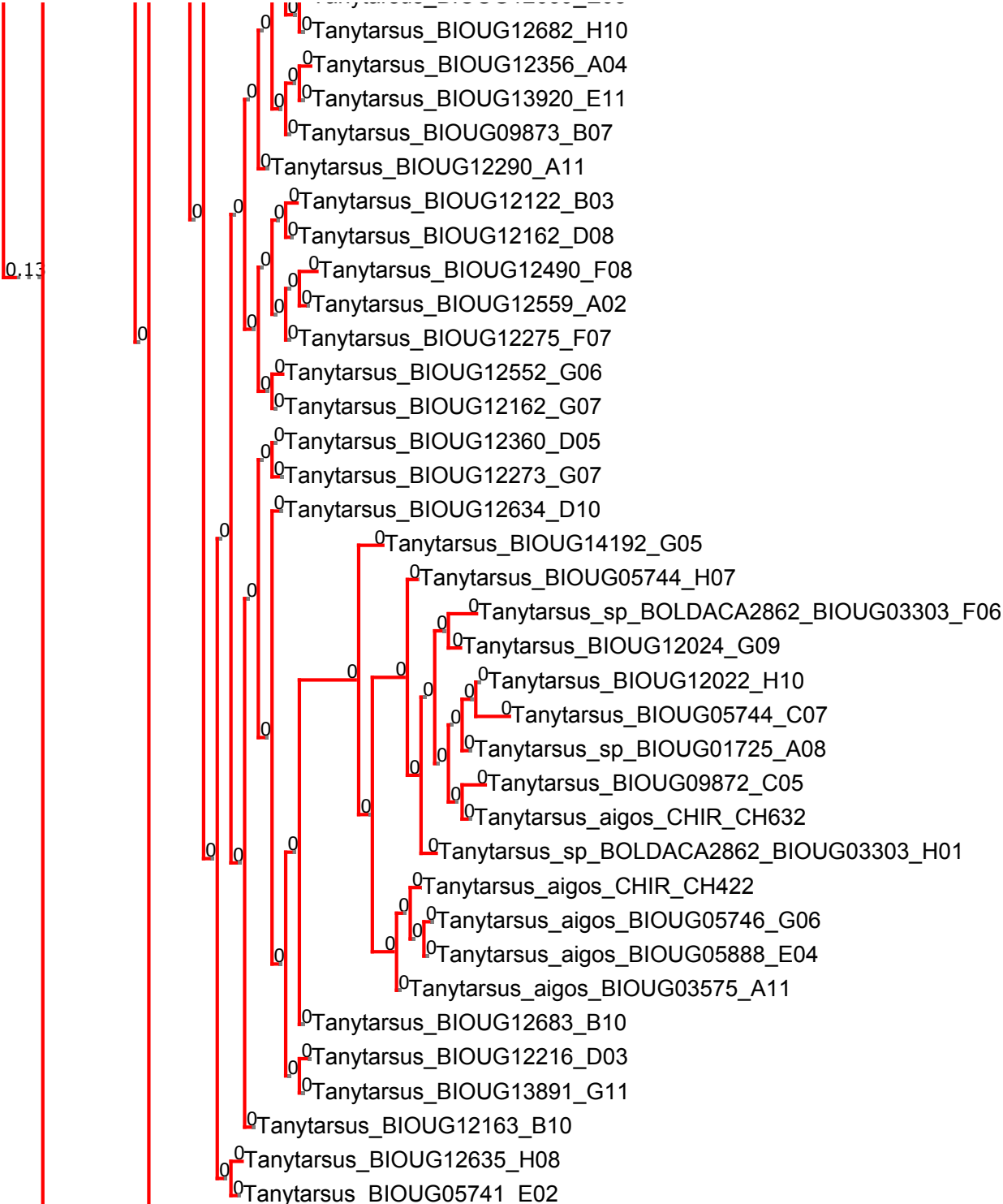

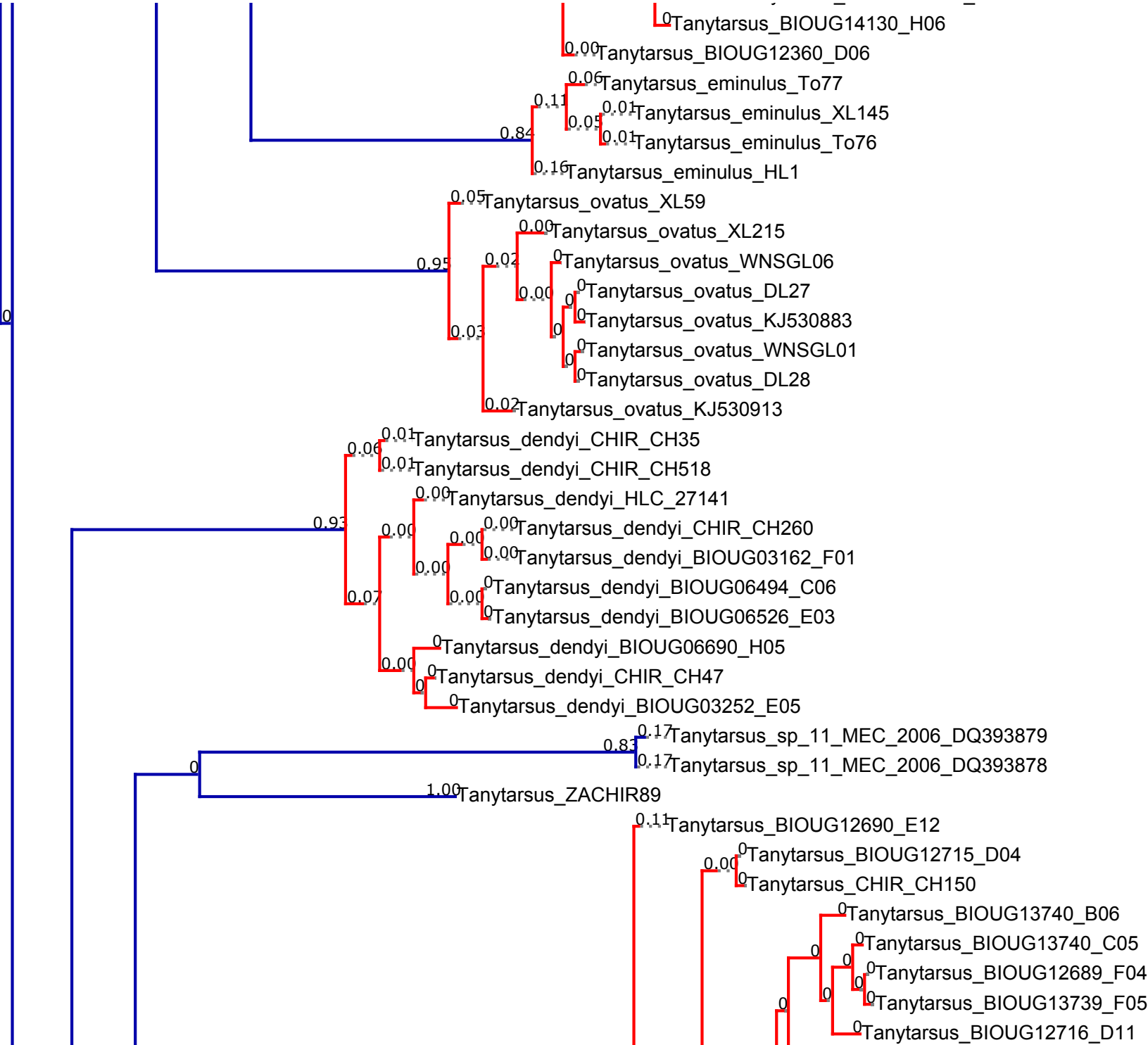

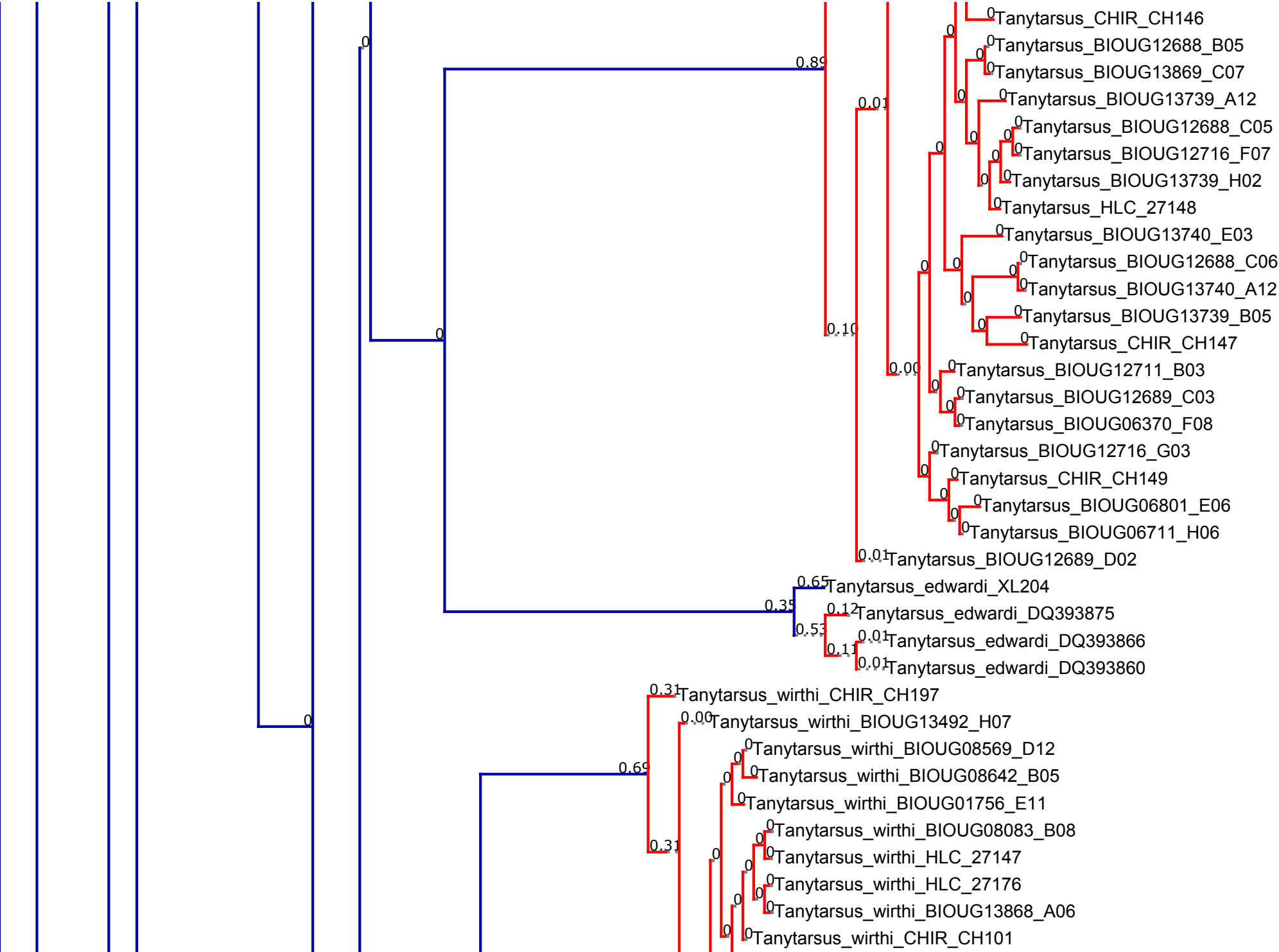

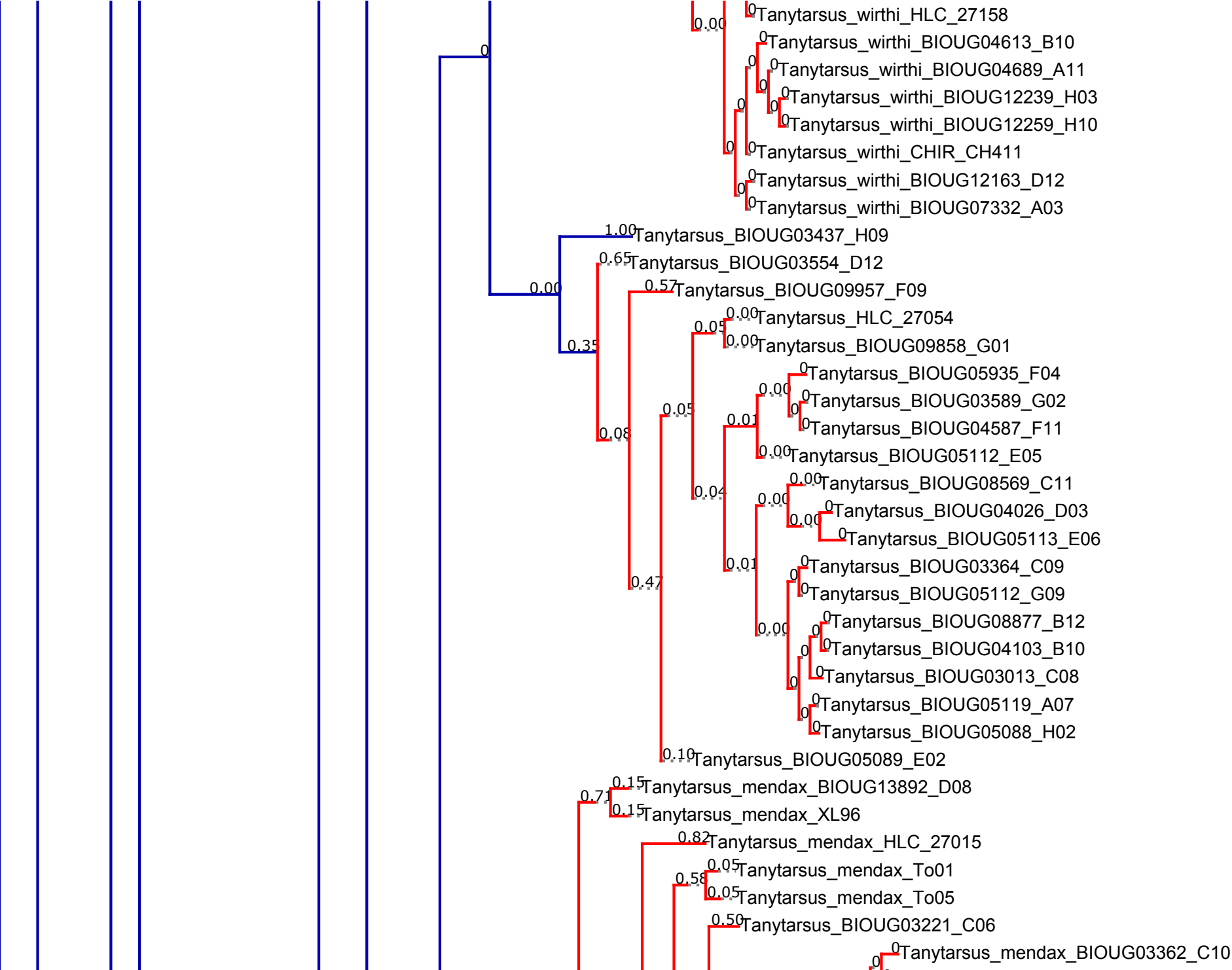

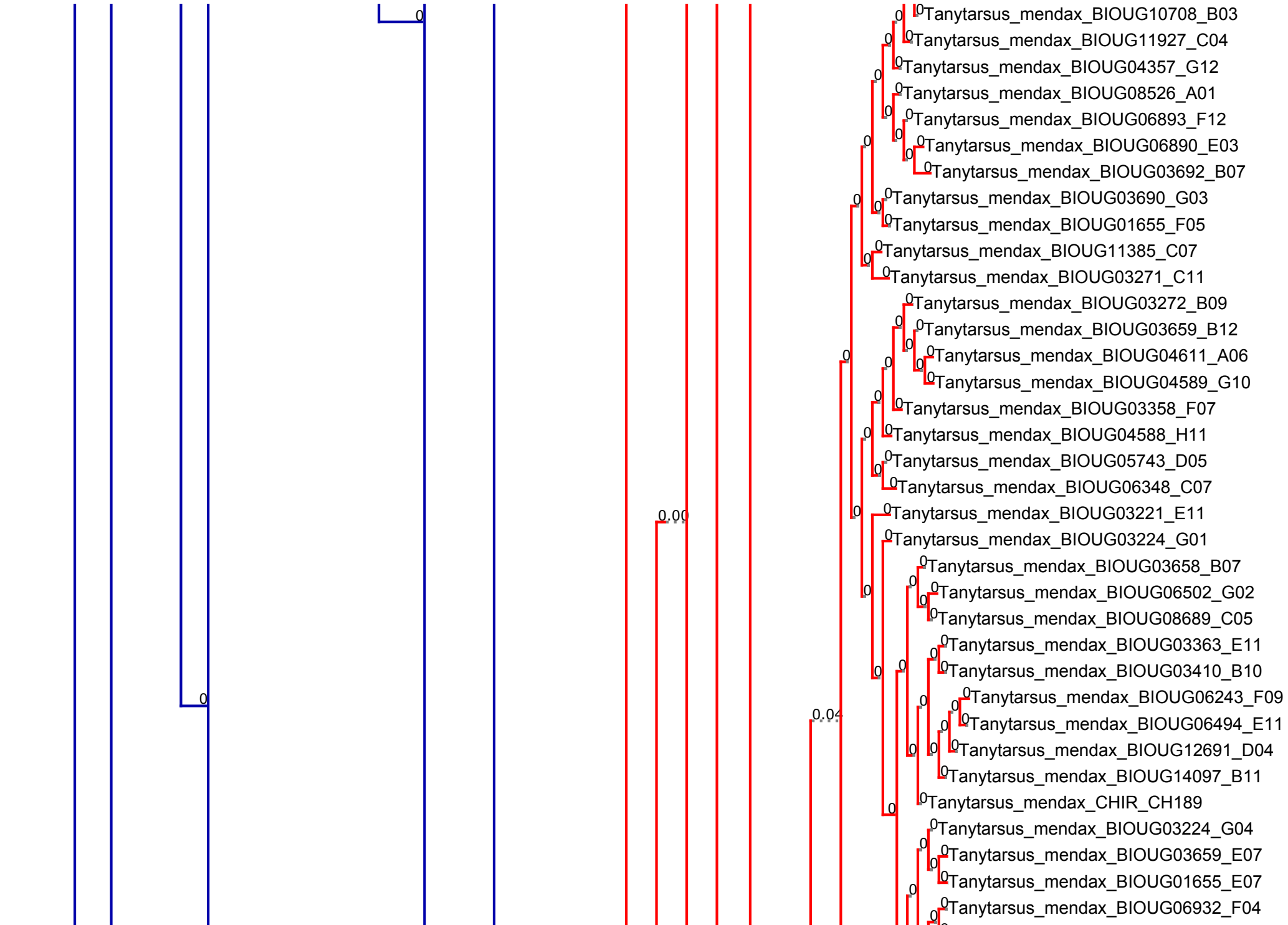

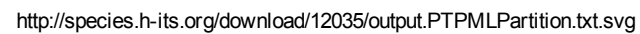

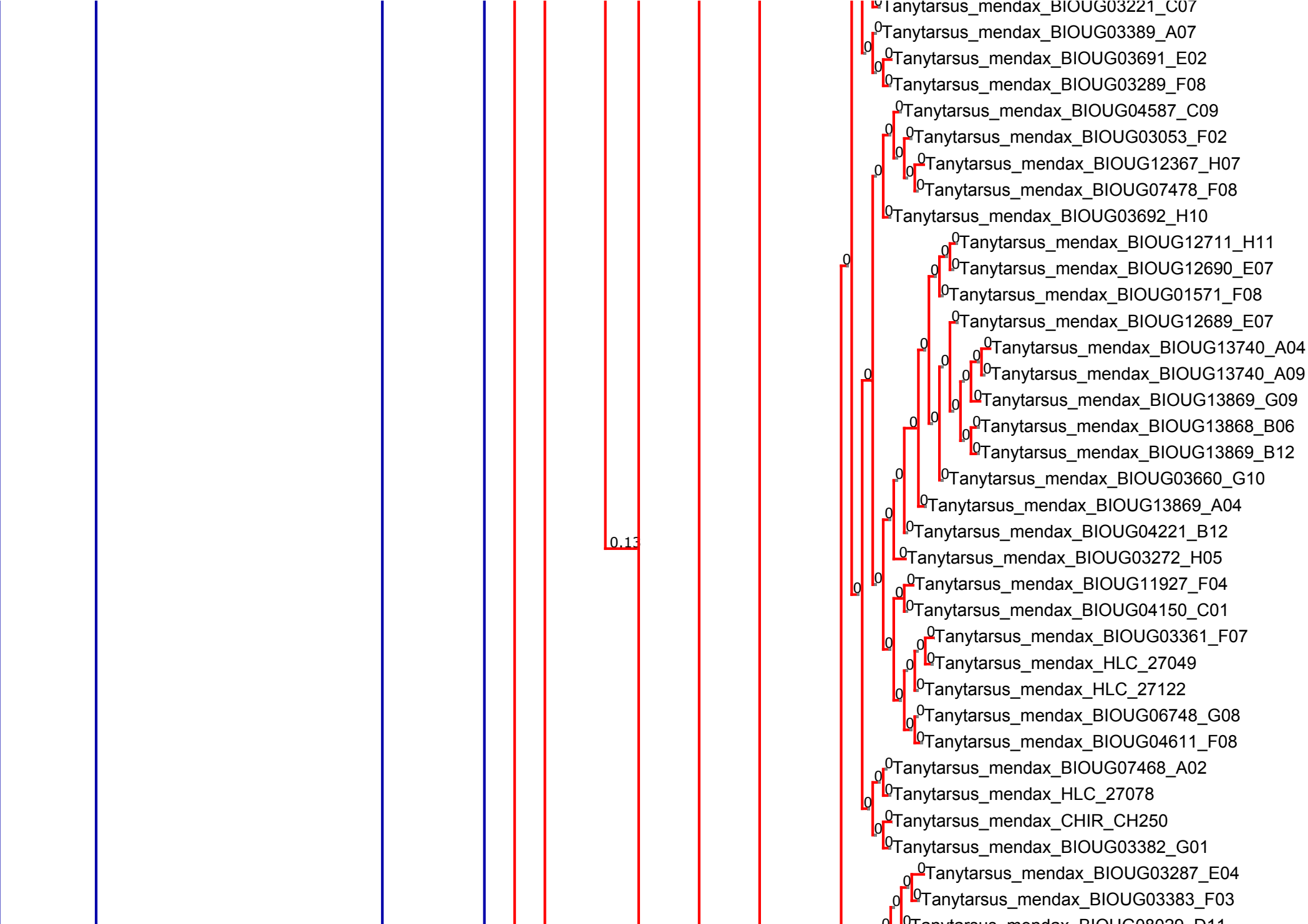

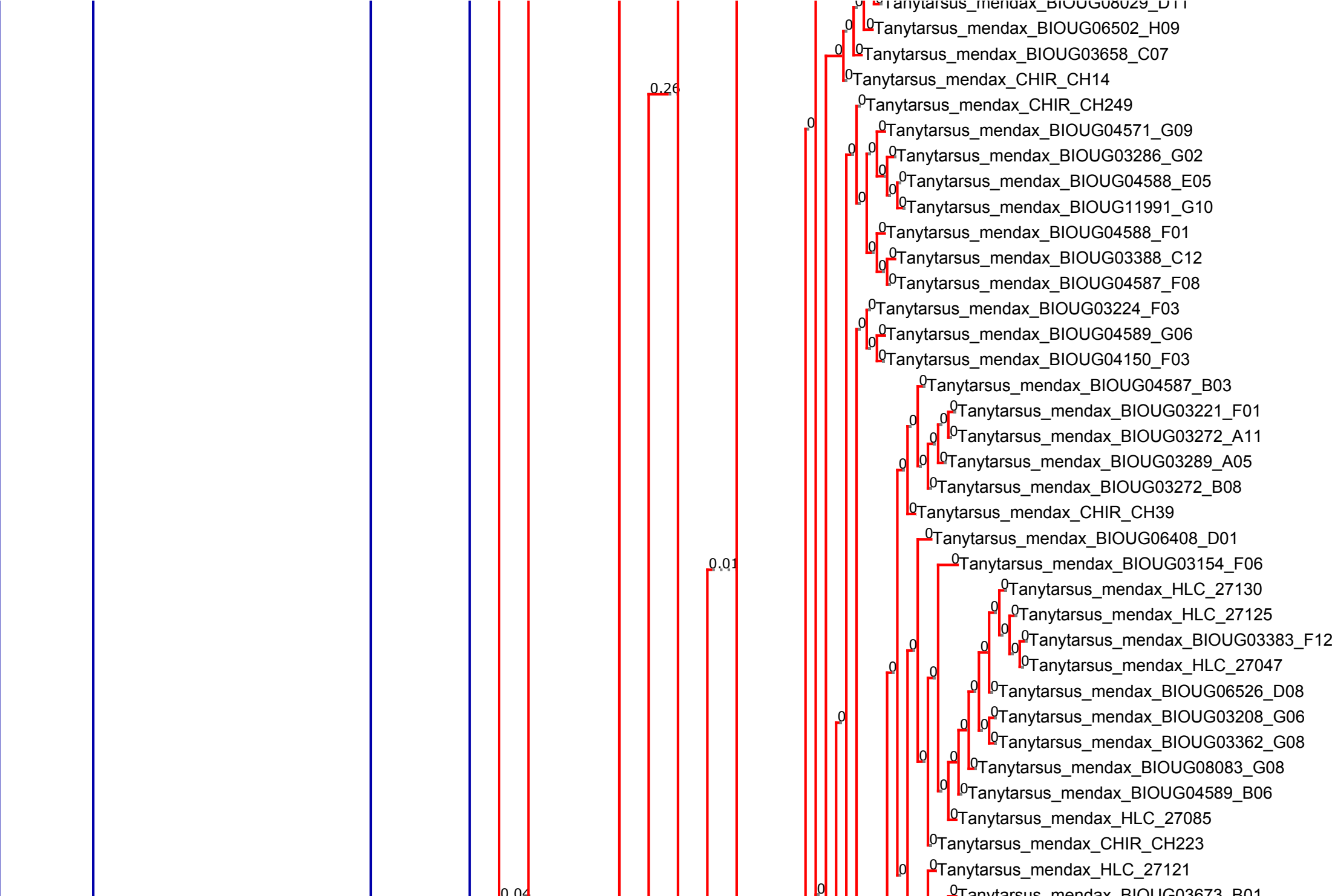

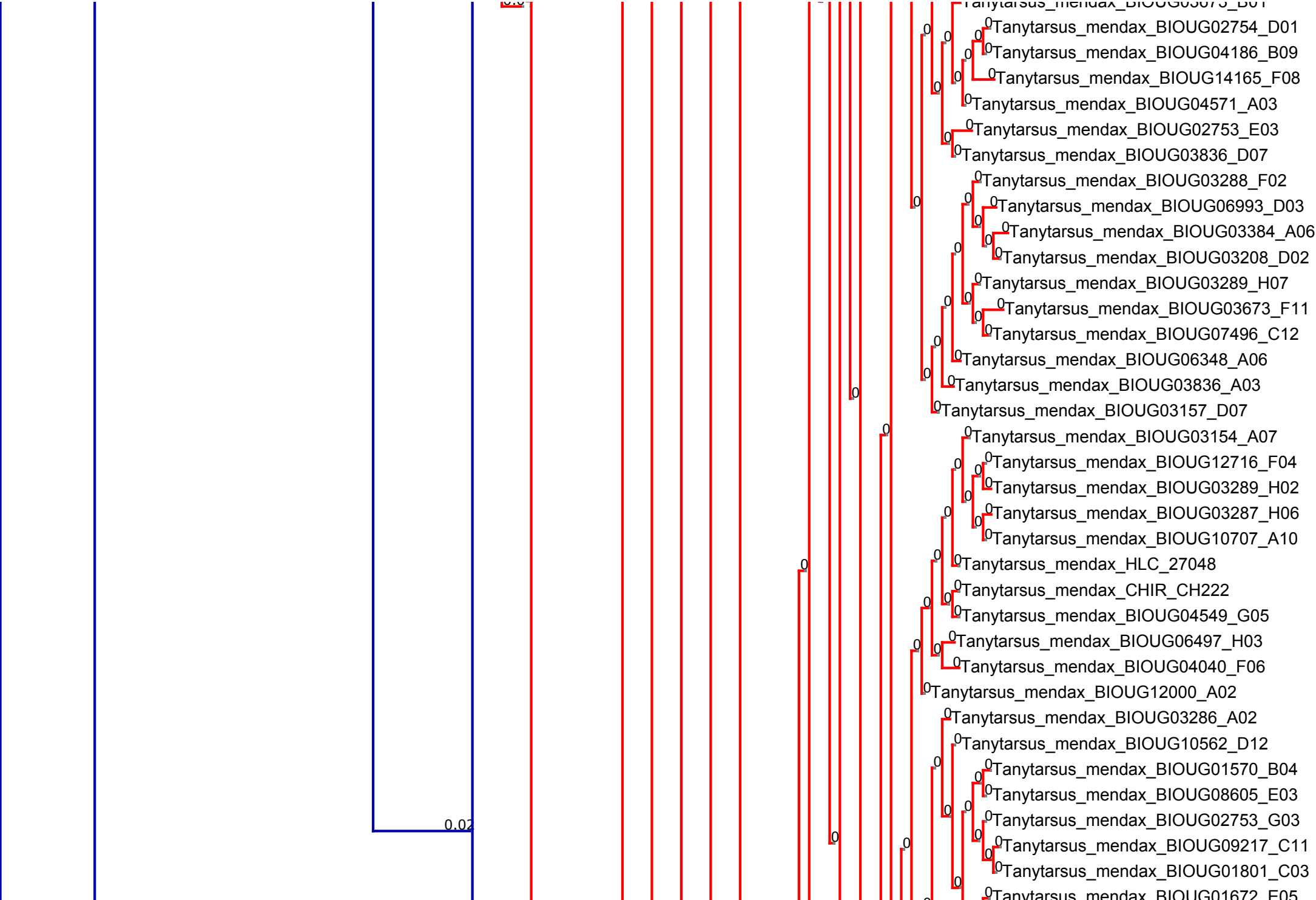

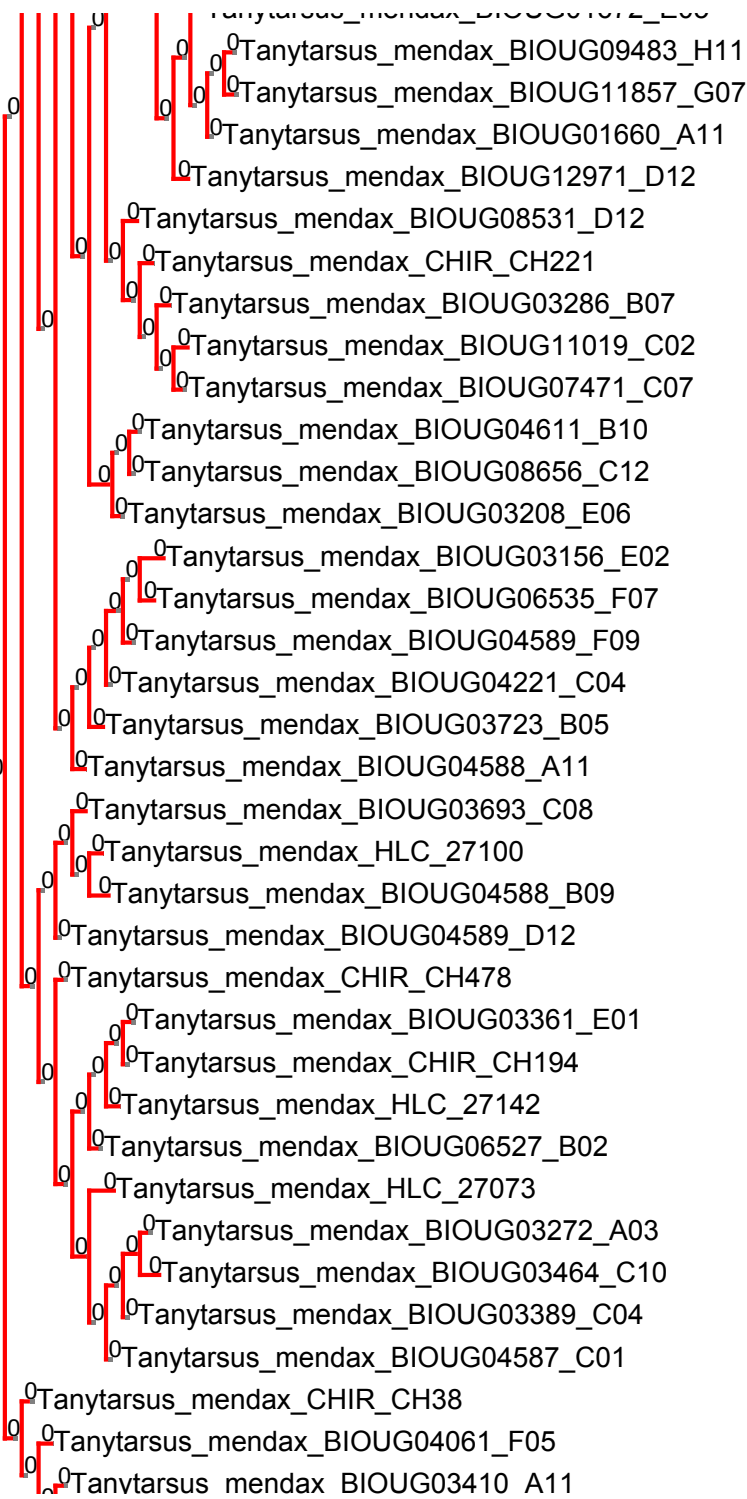

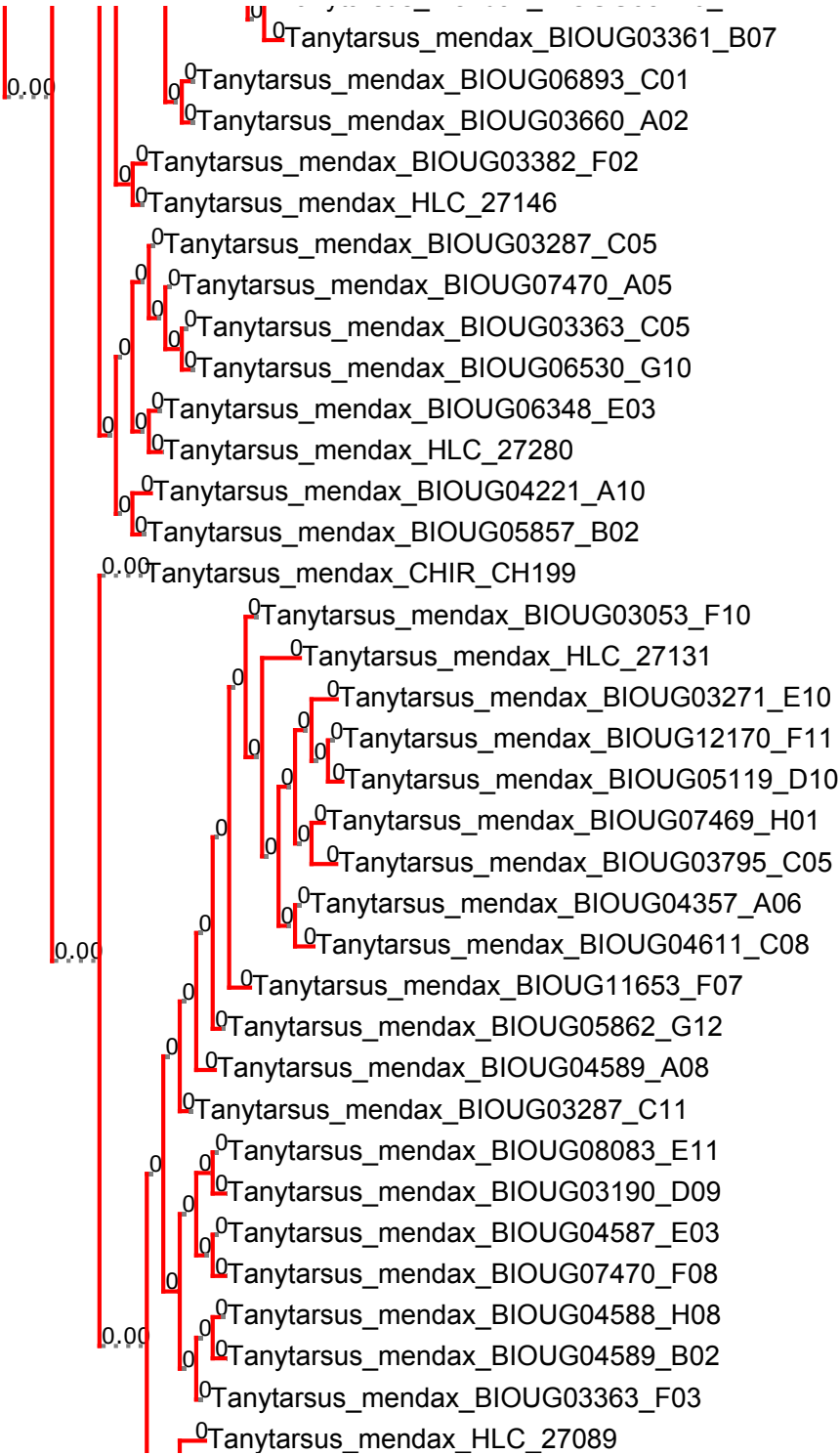

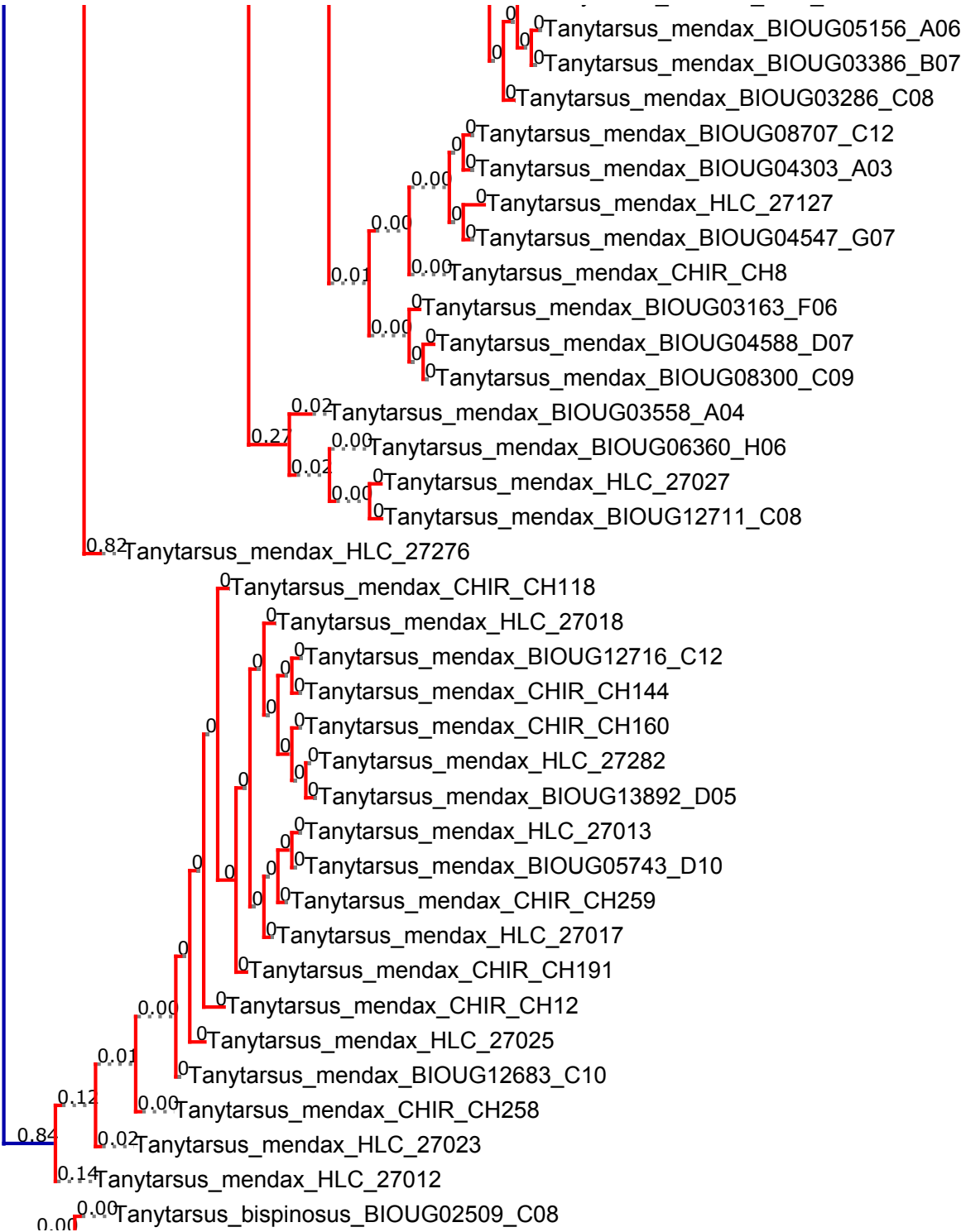

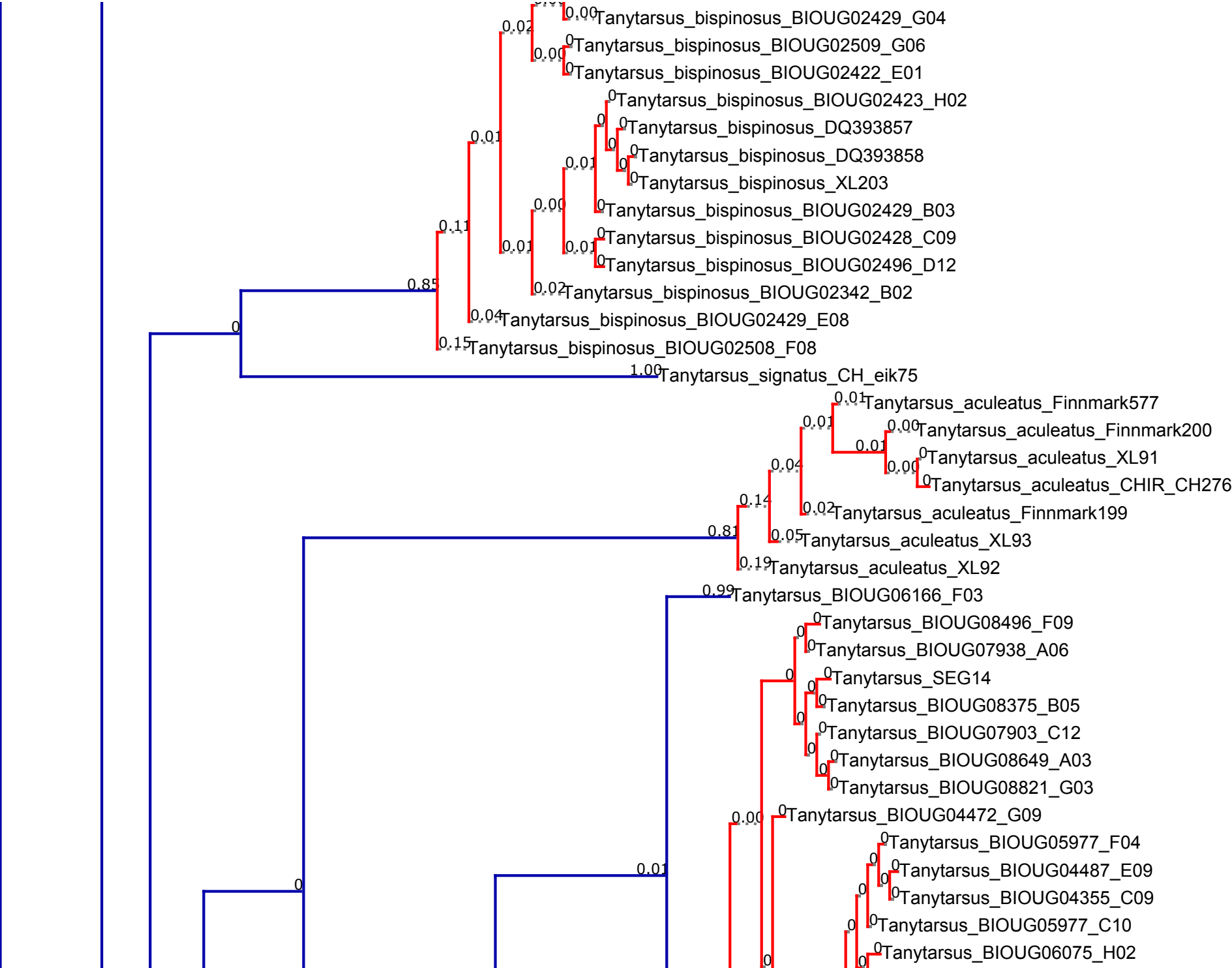

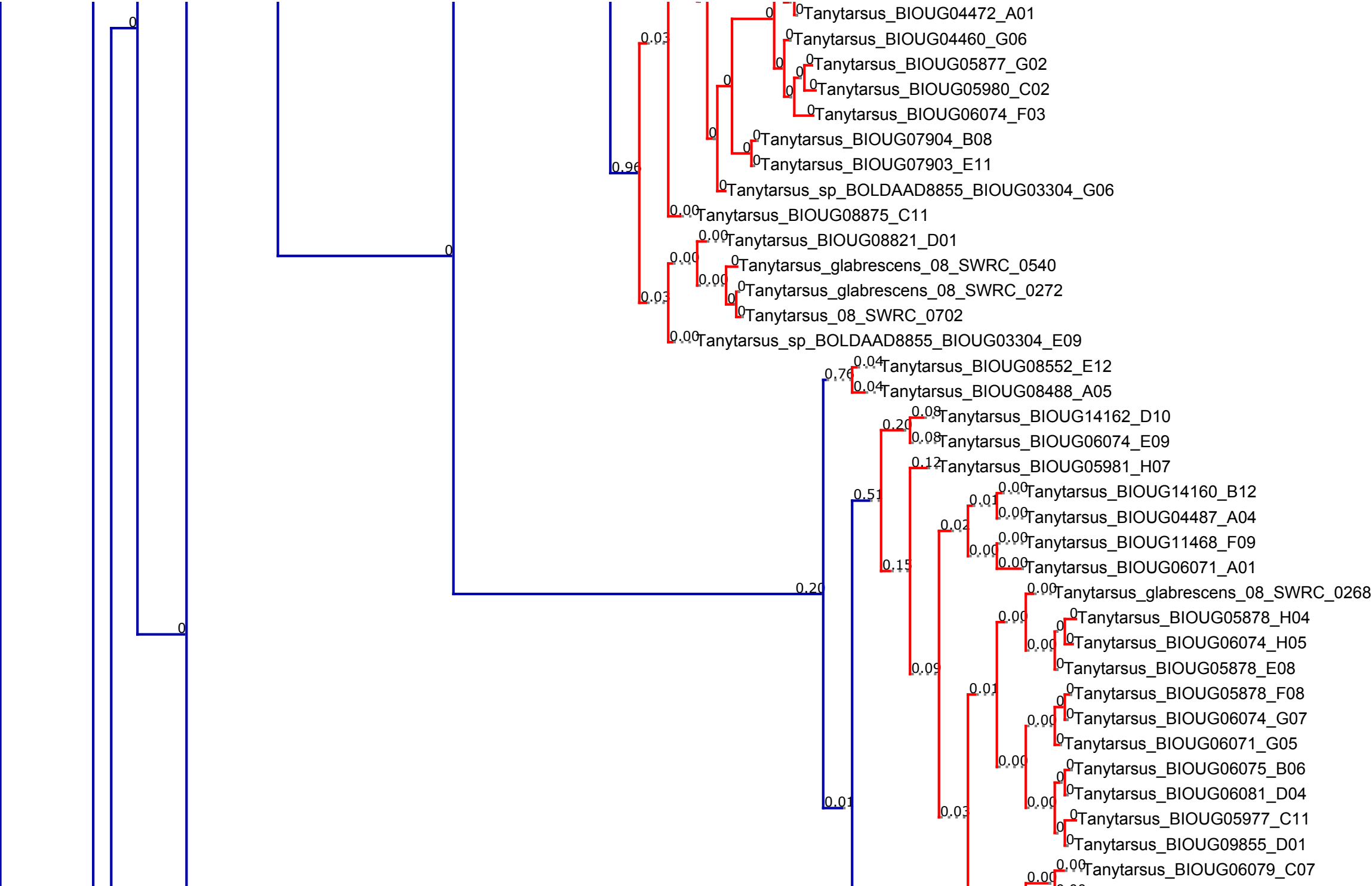

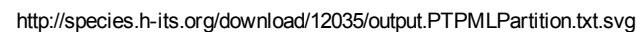

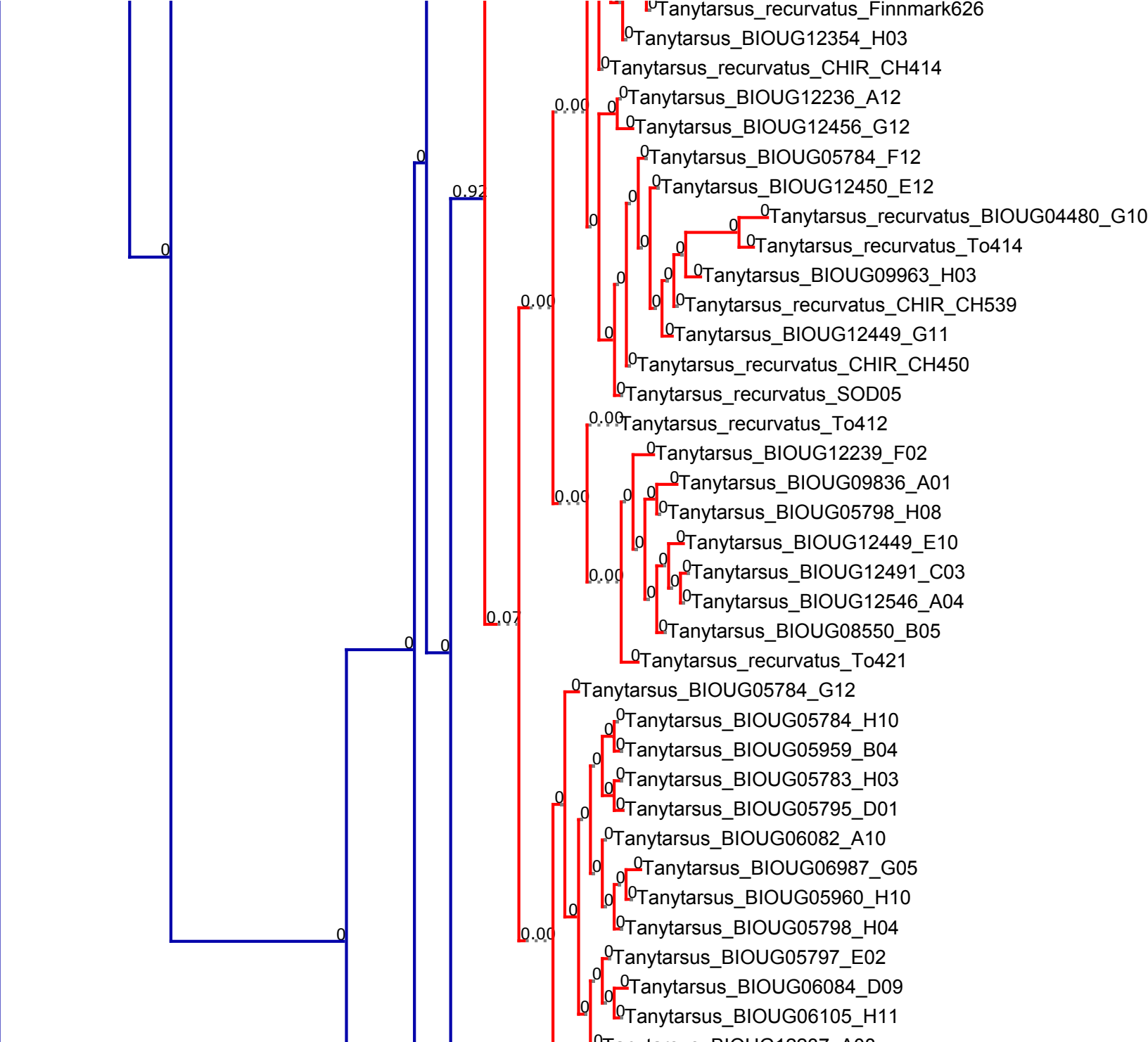

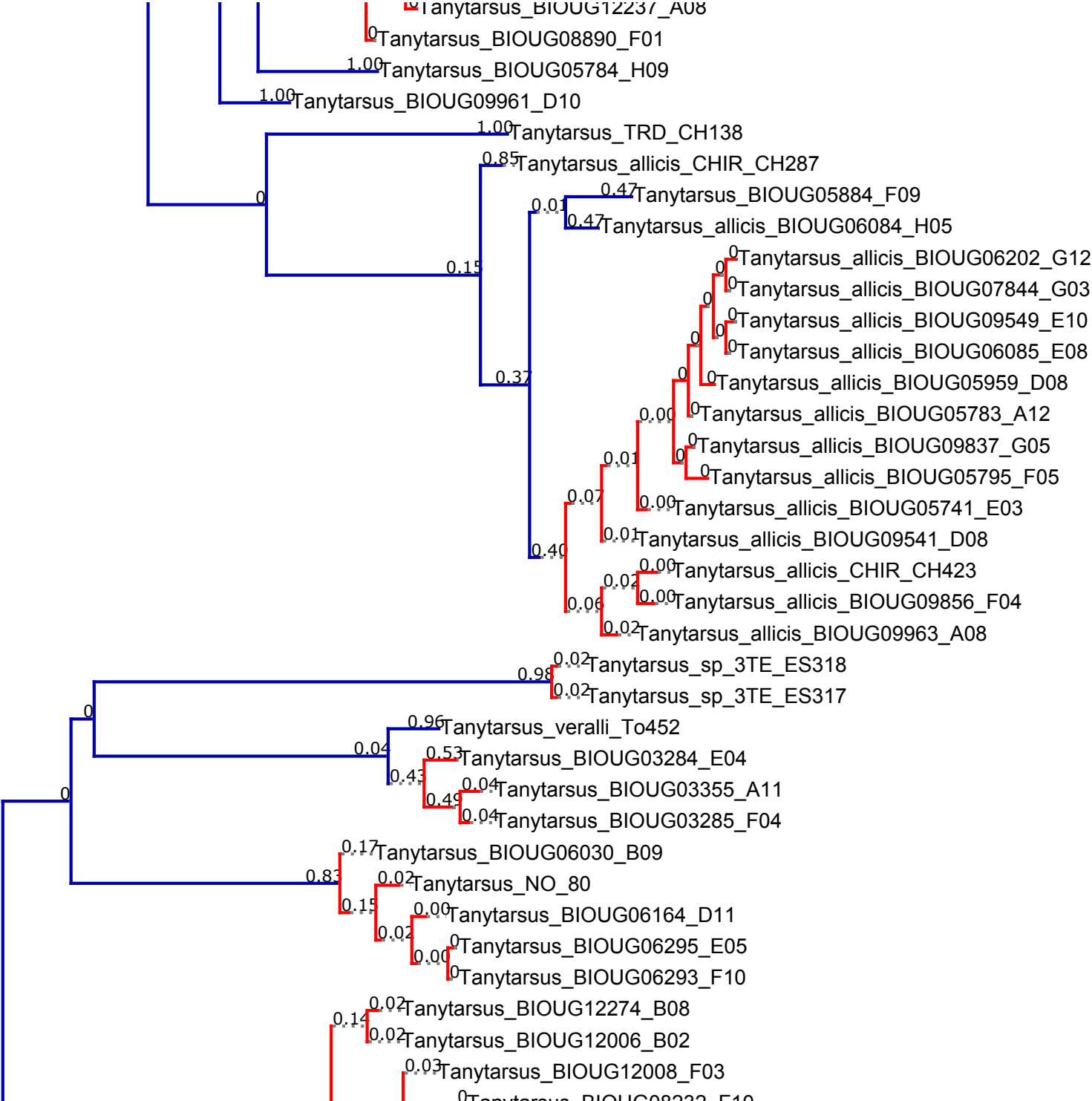

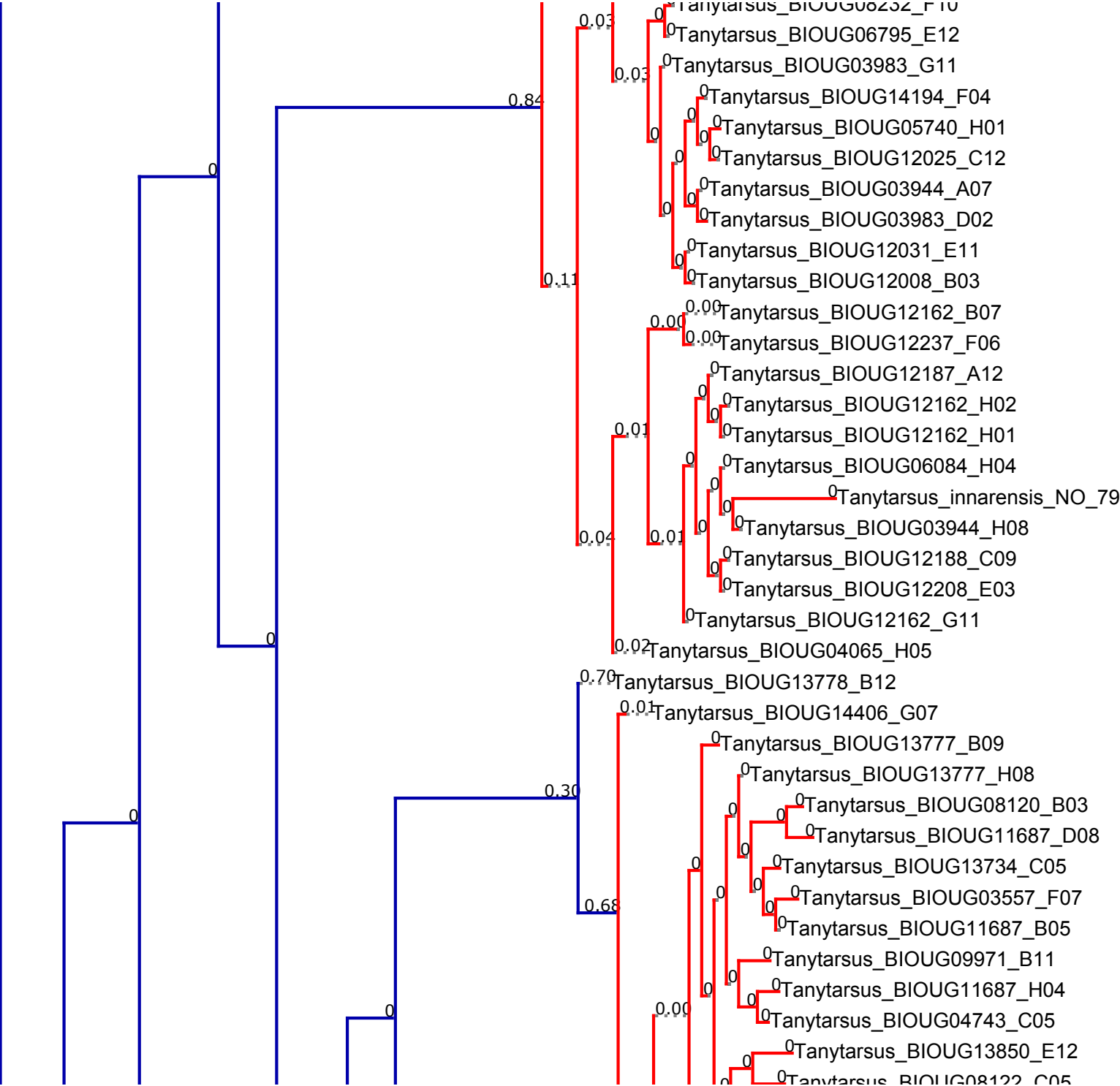

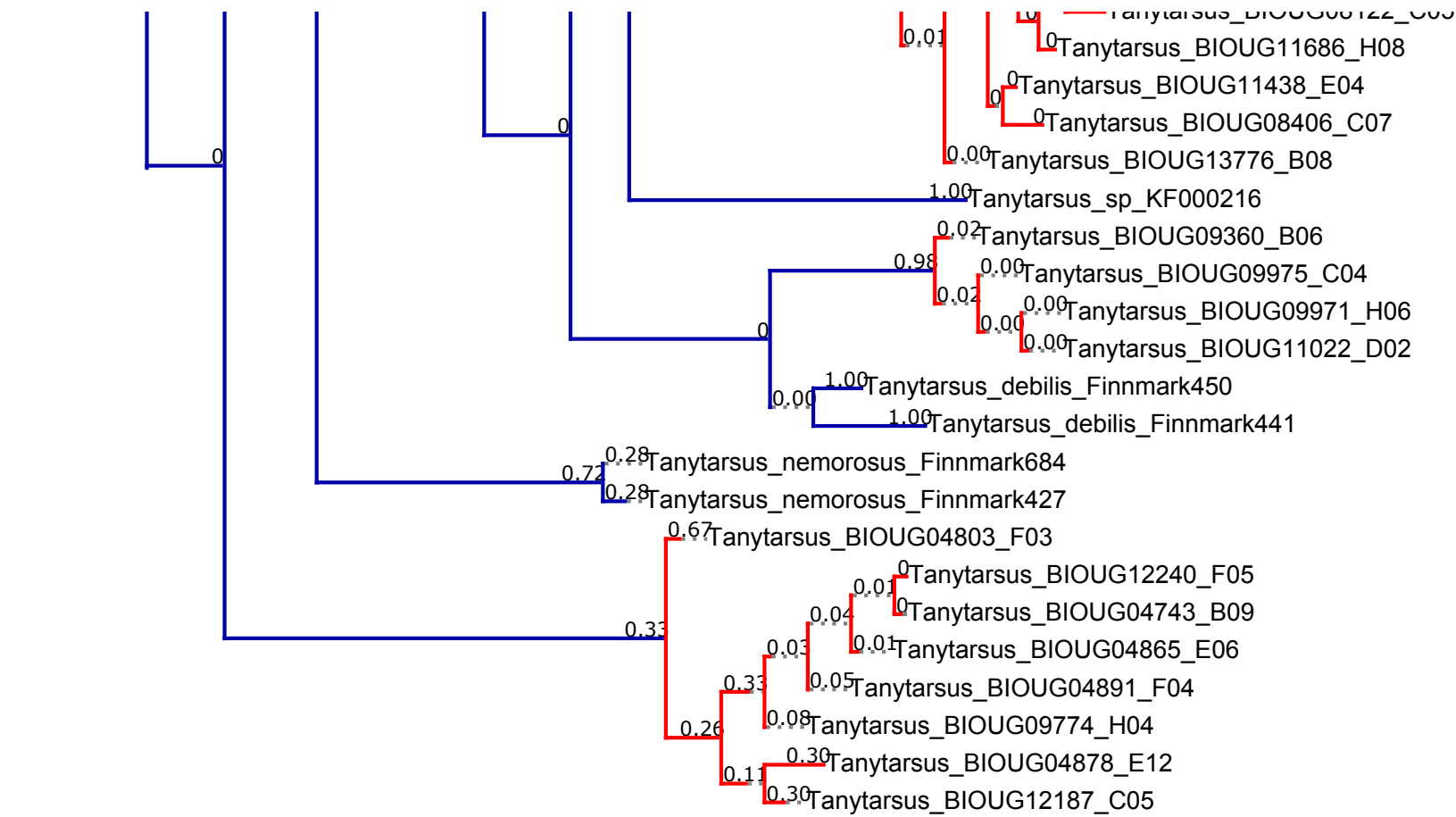

0.10

Supplement: S3 File — (PDF) [file pone.0138993.s003.PDF]
